# krepp: a *k*-mer-based maximum pseudo-likelihood method for estimating read distances and genome-wide phylogenetic placement

**DOI:** 10.1186/s13059-026-03999-y

**Published:** 2026-02-21

**Authors:** Ali Osman Berk Şapcı, Siavash Mirarab

**Affiliations:** 1 Bioinformatics and Systems Biology Graduate Program, UC San Diego, San Diego, CA 92093 USA; 2 Department of Electrical and Computer Engineering, San Diego, CA 92093 USA

**Keywords:** Metagenomics, *k*-mer-based sequence comparison, Average nucleotide identity, Phylogenetic placement

## Abstract

Comparing each sequencing read in a sample to a reference database is a fundamental step in wide-ranging applications. Results of these comparisons can enable phylogenetic characterization. However, phylogenetic placement is currently only possible at scale for marker genes, a small fraction of the genome. We introduce krepp, an alignment-free *k*-mer-based method that enables placing reads from anywhere on the genome on an ultra-large reference phylogeny (e.g., 123,853 leaves). We show that krepp is scalable and computes accurate distances that approximate those using alignments, leading to accurate placements. These precise phylogenetic identifications improve our ability to compare and characterize metagenomic samples.

**Supplementary Information:**

The online version contains supplementary material available at 10.1186/s13059-026-03999-y.

## Background

Comparing sequences of an unknown origin against an evolutionarily diverse set of reference genomes is needed in several applications, such as metagenomics [1] and contamination detection [2]. The references are evolutionarily related, and taxonomic or phylogenetic trees can be used to model those relationships. Since the genome generating a query may not be well-represented in the reference set (e.g., with the same species or subspecies), it is often insufficient to assign it to a reference genome; instead, we need to characterize it relative to all references, using a phylogenetic or taxonomic tree as a backbone. Between the two, the phylogenetic tree has a higher resolution than the taxonomy. For example, the WoL-v2 [3] phylogeny has 15,246 internal nodes compared to 3,755 in its taxonomy. A phylogeny also provides interpretable branch lengths, which can be incorporated in downstream analyses.

Constructing a phylogeny de novo using all query sequences and references is possible, but poses many challenges, such as scalability, the need for intricate pipelines, and the limited signal present in short fragmentary sequences [4]. The alternative is placing queries on an existing phylogeny, namely phylogenetic placement. Moreover, for metagenomics, placement is often sufficient as its results enable many downstream analyses [5, 6], including sample differentiation [7, 8] and UniFrac calculation [9, 10], to the extent that it has outperformed de novo phylogeny reconstruction in characterizing clinical samples [4]. Moreover, the availability of large phylogenies (e.g., [3, 11]) with tens to hundreds of thousands of leaves has provided reliable backbone trees that increase the appeal of phylogenetic placement over alternatives.

**Fig. 1:**
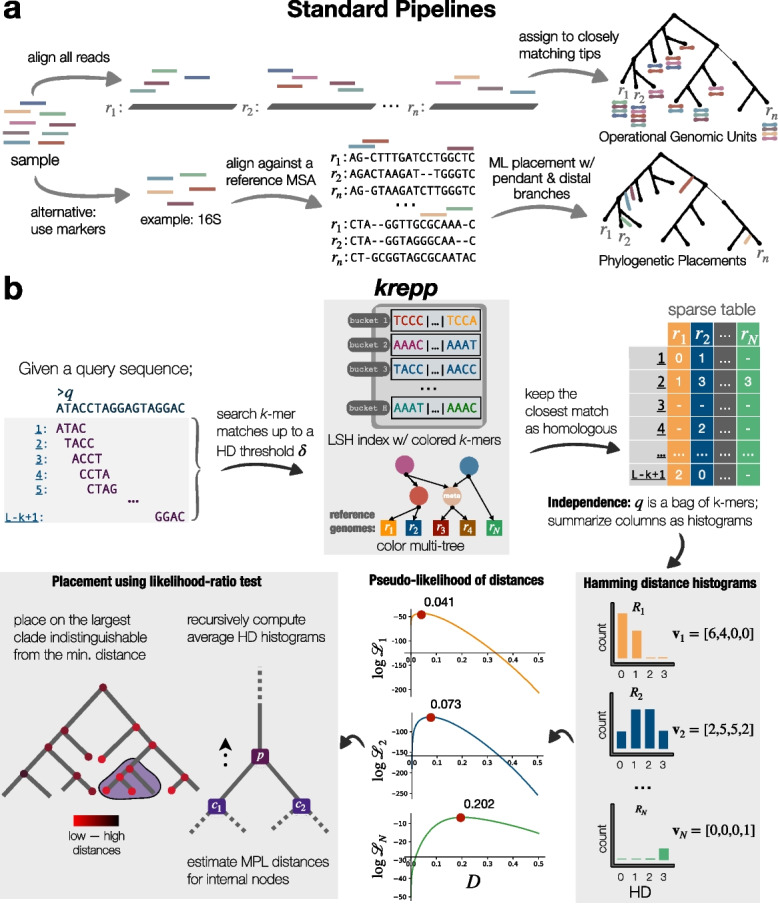
**a** Standard pipelines, either using alignment to find operational genomic units (OGUs) or mapping to marker genes for phylogenetic placement. **b** The krepp Algorithm. A query is divided into its *k*-mers, assumed independent. The first step of krepp is searching *k*-mers in the index and measuring the Hamming distance between query *k*-mers and reference *k*-mers. Due to the independence assumption, matches can be represented as histograms for each reference genome with at least one hit. Then, krepp uses its model to compute the maximum pseudo-likelihood (MPL) distances. To place on a backbone tree, distances are extended to clades, and a statistical likelihood ratio test is used to find the largest clade with indistinguishable distances from the closest match at a significance level $\alpha$. Default parameters: $\delta =4$, $k=29$, $\alpha =10\%$

Existing methods that address the ambitious goal of phylogenetic placement, often [12–18] but not always [19–21] rely on aligning queries (e.g., reads from marker genes or metagenome assembled contigs) to reference sequence alignments (Fig. 1a). As a result of relying on markers, most placement methods miss out on the vast majority of reads when the data is genome-wide (as opposed to amplification or targeted capture of marker genes). Moreover, they involve intricate pipelines with many steps, such as marker detection and (multiple sequence) alignment [17, 22], making them harder to use. An alternative is marker-free alignment-free placement of all reads, which could be done efficiently if we had reliable ways of computing phylogenetic distances between queries and genomes [23]. Computing such distances, however, is challenging for short reads. The only method that exists for phylogenetic placement of all reads (App-Spam [19]) can only handle small phylogenies, making it less useful in downstream analysis.

In contrast to phylogenetic placement, taxonomic classification and profiling methods can utilize genome-wide reads, rather than focusing on a limited set of marker genes. Many methods for taxonomy-based analysis exist (see [24, 25], for a review and benchmarking), including scalable alignment-free *k*-mer-based classification of each read (e.g., Kraken2 [26] and CONSULT-II [27]) and summarization of metagenomic samples using sketching-based [28–30] techniques (e.g., sylph [31] and sourmash [29, 32]). The scalability of these methods makes them attractive, but taxonomic classification provides less resolution than phylogenetic placement.

The only current approach that can utilize moderately-sized reference phylogenies with genome-wide data is an idea called operational genomic units (OGUs) [33]. OGUs are obtained by aligning reads to reference genomes and retaining matches with high sequence identity. These OGUs can then be summarized into taxonomic profiles or assigned to leaves of a backbone phylogeny (e.g., the method Woltka [33]). Nevertheless, this approach does not model phylogenetic relationships between references to guide the mapping stage, and fails to scale to ultra-large references due to the computational demands of the read-to-genome alignment step.

The goal of this paper is to develop a scalable method that can combine the benefits of phylogenetic placement with those of alignment-free read characterization. More specifically, given a large set of query reads of unknown origin (e.g., a metagenomic sample), and a large and evolutionarily diverse set of reference genomes $\mathcal {R}$: *i*) We seek to *compute the distance* from each read *q* to all reference genomes $r\in \mathcal {R}$ that are sufficiently close to *q*. *ii*) When also given a reference phylogeny *T* leaf-labeled by $\mathcal {R}$, we seek to *place each query read* independently onto branches of *T*.

We define the read-genome distance as the Hamming distance between the source genome of *q* and a reference genome, restricted to the region from which *q* is sampled. If the rates of evolution were fixed across the genome, this would be $1-$ANI (average nucleotide identity), but since rates can vary, the read-genome distance matches $1-$ANI only in expectation. We primarily focus on the case of short ($\approx 150$bp) reads for which the best existing approach to compute such distances is aligning *q* to reference genomes. At this length, the signal is insufficient for MinHash-based [34] methods (e.g., MashMap [35]) to perform well. While efficient aligners such as bowtie2 [36] and minimap2 [37] exist, they do not scale well to tens of thousands of reference genomes and are less effective at high distances ($>10\%$).

We propose a scalable solution to both problems—a *k*-mer-based algorithm to compute the distance between a short query sequence and relevant reference genomes and a placement method. The resulting method, krepp (*k*-mer-based read phylogenetic placement), is scalable to tens of thousands of microbial reference genomes and is accurate both in terms of distances it computes and its placements.

## Results

### From *k*-mers to alignment and placement

We propose an algorithm to *i*) compute the distances between a read and a set of reference genomes and *ii*) use the results for phylogenetic/taxonomic placement (Fig. 1b). The distance calculation is the fist algorithmic stage, which has two aspects: indexing *k*-mers from reference genomes in a way that allows inexact matches at the query time, and computing distance given inexact *k*-mer matches. We start by giving a high-level overview of each algorithmic component, and then expand on each technique in Methods, using the notation in Table 1 throughout.

**Table 1:** Notations used throughout the paper

Symbol	Definition	Symbol	Definition
$\mathcal {R}$	Set of all references	$\mathcal {R}(x) \subseteq \mathcal {R}$	Set of genomes that include a *k*-mer *x*
$\mathcal {M}$	Set of all *k*-mers	$\mathcal {M}(t) \subseteq \mathcal {M}$	Set of *k*-mers of all genomes under node *t*
*T*	Reference tree with $\mathcal {R}$ as leaf set	C	A color, $\mathcal {R}(x)$ for some $x\in \mathcal {M}$
$\delta$	Maximum HD for *k*-mer matches	$\mathcal {C}$	Set of all observed colors: $\{\mathcal {R}(x) : x \in \mathcal {M} \}$
*h*	Positions sampled for LSH	$\textbf{A}, \textbf{C}$	LSH sorted arrays of *k*-mers and color IDs
$\tau$	Match count threshold for placement	$\textbf{G}$	Color multitree $G=(V,E)$, $\mathcal {C} \subseteq V$
$\rho _r$	Subsampling rate for reference *r*	$\textbf{I}$	Offset-index array for bucket boundaries
$u_r$	Number of mismatches to ref. *r*	$v_{r,d}$	Number of matches at HD${=}d$ to ref. *r*

#### Indexing and *k*-mer matching

To increase sensitivity and enable precise calculation of high read-to-genome distances, we allow query *k*-mers to match similar reference *k*-mers, measured by the Hamming distance (HD). We achieve this by partitioning reference *k*-mers (default: 29-mer minimizers of 35-mers) into buckets using locality-sensitive hashing (LSH), a scheme adapted from the CONSULT family of methods [27, 38, 39] with substantial changes required for distance calculations. We use the bit sampling technique to compute hash values by selecting *h* (default: 14) random but fixed positions from *k*-mers, resulting in $2^{2h}$ buckets, which together constitute the LSH index. Then, during the query time, we compute the HD between a query *k*-mer and all reference *k*-mers in the bucket specified by its hash value, and retain matches up to an HD threshold $\delta$ (default: 4) as its putatively homologous counterparts in reference genomes.

#### Colored *k*-mer representation

After finding *k*-mers in the LSH index that are similar to a query *k*-mer, we need to identify reference genomes that include those *k*-mers, which is the well-studied colored *k*-mer representation problem [40]. Here, *color* refers to a subset of reference genomes with at least one shared *k*-mer (a singleton genome is also a color). The literature primarily focuses on highly similar reference genomes (e.g., pangenomes) in the context of colored de Bruijn graphs [41–43]. In our application, *k*-mers come from an evolutionarily diverse set, making most (but not all) colors sparse (i.e., the vast majority of the colors are expected to include a small portion of the references). We represent each non-singleton color as a union of other colors. This effectively defines a directed acyclic graph (DAG) $G=(V, E)$, with colors as nodes and edges representing set partitioning. A trivial DAG makes each non-singleton color the parent of all its associated singletons (i.e., leaves of the DAG). To build a more compact DAG (w.r.t. $|E|+|V|$), we use the insight [44] that for similar genomes, we expect to see highly overlapping colors, which can be captured by adding “meta”-colors that represent the shared genomes (see Additional file 1: Fig. S1). Finding a minimal DAG has been an intractable problem and has motivated heuristic solutions such as clustering [44]. Instead, we utilize the given phylogeny and gradually build a multitree of colors using a post-order traversal, updating existing colors and adding new ones when needed at each node.

#### Maximum pseudo-likelihood distance estimation

Searching read *k*-mers against the LSH index and color multitree gives us the set of putatively homologous *k*-mers in each reference genome, from which we compute distances. Considering the difficulty of modeling the dependencies between overlapping *k*-mers (which is only studied in the context of exact matches [45]), we ignore the *k*-mer positions and assume *k*-mer independence. This assumption allows us to summarize homologous matches against a reference genome *r* as a histogram, denoted by $\textbf{v}_r=(v_{r,0}, \dots , v_{r,\delta })$, where $v_{r,d}$ is the number of matches of Hamming distance *d* to the reference *r*, and the number of mismatches $u_r$, which is simply $L-k+1-\sum _{d=0}^{{\delta }}v_{r,d}$, where *L* is the read length. For a reference *r*, each query *k*-mer is either a match at an HD${=}d$ or a miss against all indexed *k*-mers of *r* with *k*-mer subsampling rate, $\rho _r$, which is given by the ratio of selected minimizers to the total number of distinct *k*-mers. Thus, the likelihood of having the read-wide distance *D* can be written as a product over all query *k*-mers: $\mathcal {L}_r(D;k,h,\delta ,u_r,\textbf{v}_r,\rho _r)=\left( P_{\textrm{miss}}(D;k,h,\delta ,\rho _r)\right) ^{u_r}\prod _{d=0}^\delta \left( P_{\textrm{match}}(D;d,k,h,\rho _r)\right) ^{v_{r,d}}$ where $P_{\textrm{match}}$ is the probability of observing a match of HD *d* and $P_{\textrm{miss}}$ is the probability of no match up to the HD $\delta$. We compute these probabilities (see Methods) using a simple substitution model and the mathematical properties of LSH. Optimizing the logarithm of this single variable likelihood function, $\mathcal {L}_r$, results in point estimates for distances between each query read and every reference with at least one match (see three examples in Fig. 1b).

#### From distances to phylogenetic and taxonomic placement

Beyond outputting all read-to-genome distances, krepp can characterize a read by finding all statistically indistinguishable best matches (similar to OGUs), placing the read on a given tree *T* (including on internal nodes), or classifying it taxonomically. Employing a likelihood-ratio test (i.e., the $\chi ^2$ test with 1 degree of freedom) to account for noise in estimates, we can find all distances that are statistically indistinguishable from the minimum distance of *q* to any node. To allow phylogenetic placement, we extend distances to clades. To motivate our approach, note that for an ultrametric tree, a principled approach is to place *q* as the sister to the largest clade where all leaves have distances that are indistinguishable to the minimum distance (Additional file 1: Fig. S2a). Extending on this intuition, our algorithm requires a rooted tree and recursively defines distances from reads to clades in a bottom-up fashion by summarizing *k*-mer histograms at higher levels (see Methods). Among all clades (including leaves) with statistically indistinguishable distances, we place the read as a sister to the largest clade, breaking ties by choosing the clade with the smallest distance. We leave *q* unplaced if the chosen clade is the root or there are too few *k*-mer matches (see Methods).

For taxonomic classification, one option is assigning each query the same taxonomic label as the reference with the minimum distance (krepp-closest hereafter). Since this approach ignores the taxonomic tree, we also directly apply the phylogenetic placement algorithm to the multifurcating taxonomic tree, marking “species” as the tree leaves (referred to as krepp-placement). To compute krepp distances to each species, represented by one or more genomes, we compute the average Hamming distance histogram ($\textbf{v}$), mismatch count (*u*), and subsampling rate ($\rho$) for all genomes corresponding to it. With these average statistics at hand, and noting that the krepp algorithm does not need a binary tree, we place each query independently on the taxonomic tree, each branch of which corresponds to a taxonomic label at some rank.

### Benchmarking on simulated reads

#### krepp accurately estimates read-to-genome distances

**Fig. 2:**
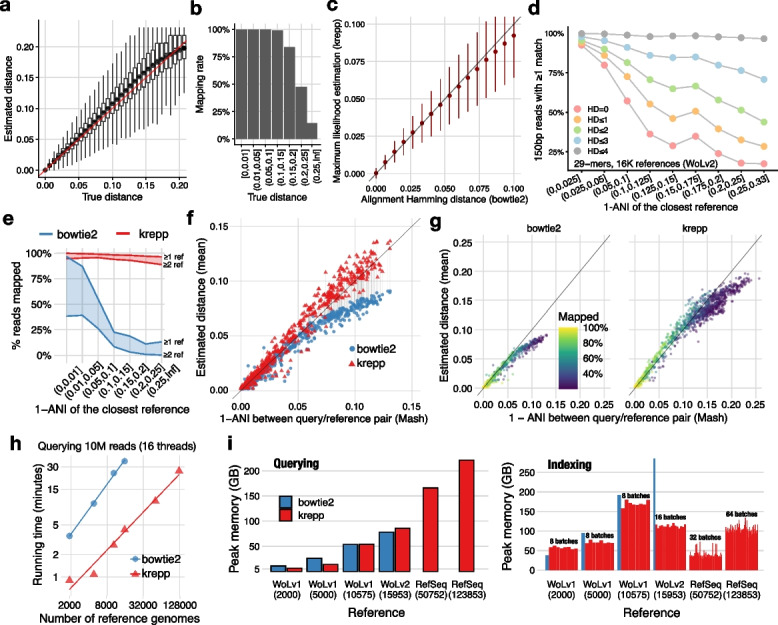
**a** Comparing the true Hamming distance (normalized by the read length) and the estimated Hamming distance for individual 150bp short reads (1M total). Each distribution is over 2,500–50,000 reads. **b** Average percentage of reads mapped in each true distance bin. **c** MPL distance estimate of krepp versus bowtie2 alignment distance, i.e., HD normalized by the read length (mean, standard deviation computed over a subsample of 1M reads). **d** As the genome-wide distance, estimated using Mash [48], between the query genome and the closest reference increases, fewer 150bp short reads have any 29-mer (minimizers of 35-mers) that match a reference exactly, but most have some at HD 4. **e** The portion of reads mapped to at least 1 (top) or 2 (bottom) references, binning queries by the genome-wide distance to their closest reference. We use bowtie2 with its --very-sensitive and --all options, and krepp with its defaults. **f** Mean estimated distance across reads for each query/reference genome pair (connected dots) where $\ge$20% of reads are mapped by both methods. *x*-axis: genome-wide distances between the query/reference pair ($D^*$); *y*-axis: mean Hamming distance across all reads mapped to a reference genome ($\bar{D}$). **g** $\bar{D}$ versus $D^*$ from each query to all WoL-v2 references with at least 20% reads mapped (colors) for each method. **h** Running time (log-log scale) of querying 10M short reads versus the number of references. Line slopes imply $\mathcal {O}(n^{0.92})$ and $\mathcal {O}(n^{1.09})$ growth for krepp and bowtie2 on these datasets. Measurements were performed on 2.25 GHz AMD EPYC 7742 CPUs using 16 threads and 256GB DRAM. **i** Memory requirements of krepp, compared to bowtie2 for reference datasets with varying sizes (*x*-axis: 2000–123,853 genomes), both for indexing (right) and querying reads (left). krepp can build its index in batches (shown as separate bars), controlling the peak memory use, whereas bowtie2 fails to index the two largest reference sets as its peak memory usage exceeds 256GB

We first benchmarked krepp’s MPL distances in simulations with known ground truth. We used simulations from Stepanauskas et al. [46] who mutated 29 base genomes from Pachiadaki et al. [47] at controlled distances using a sequence evolution model with rate heterogeneity. We generated 150bp short reads from simulated genomes without errors. On these simulated data, krepp achieves high accuracy and very little bias in computing the true read-to-genome distance (Fig. 2a). As expected, there is noise, and the noise increases for higher distances (especially beyond 10%). The mapping rates exceed 80% even for reads with distances up to 20% and are close to perfect for less distant reads (Fig. 2b). The krepp estimates also accurately approximate the genome-wide mean and standard deviation of distances (Additional file 1: Fig. S3).

Since our simulations are simple compared to real data, we next used 15,953 real microbial genomes from WoL-v2 as reference and selected a separate set of 500 query genomes, spanning a wide range of novelty levels measured by the distance (1-ANI) to the closest reference genome (Additional file 1: Fig. S4b). We computed the genome-wide distances between each query and all reference genomes using Mash [48] and refer to it as $D^*$. We simulated 33 million 150bp reads across all query genomes with the default Illumina error profile, and computed read-genome distances (*D*) using bowtie2 run with high sensitivity and looking for all matches.

For reads with distance $D\le$10%, where bowtie2 tends to be accurate, we take its output as ground truth. In these cases, krepp computes similar distances to bowtie2 (Fig. 2c). As in simulations, variance increases with distance, but estimates show little sign of bias. An advantage of krepp compared to alignment (e.g., using bowtie2) is that it maps more reads due to highly sensitive LSH-based search (Fig. 2d). Compared to exact *k*-mer matches, our indexing scheme boosts the portion of reads with at least one hit up to fourfold for relatively novel query genomes (>10% distance to the closest match) with respect to the reference set. In particular, for those in the 10–20% distance range, only 30% of reads have an exact match, but more than 80% have inexact matches with HD$\le 3$. More than 98% of reads are mapped to at least one and often multiple genomes using krepp, compared to 39% for bowtie2. The higher mapping rates of krepp are mostly for novel genomes (Fig. 2e). Binning query genomes by the novelty, we observe that bowtie2 fails to match 84% of the reads to *any* reference when novelty exceeds 10%, whereas krepp still maps 98% of reads to at least one reference for these novel queries (these portions include reads mapped to reference genomes other than the closest match).

Notably, the high-distance matches from krepp stay accurate. Since we cannot rely on alignment results to get the ground truth for highly distant reads, to probe accuracy, we rely on genome-wide averages. We compute the mean *D* across all reads mapped to a reference genome ($\bar{D}$). Focusing on query/reference pairs where at least 20% of reads are mapped by both methods, when $D^*<5\%$, both bowtie2 and krepp are accurate, with bowtie2 performing slightly better (Fig. 2f). However, as $D^*$ increases to 5–12%, the bowtie2 average severely underestimates the Mash distance (1.3$\times$ on average) while krepp overestimates it to a lesser degree (1.09$\times$ on average). Examining all pairs where *each* method maps at least 20% of reads, krepp distances remain accurate on average even for $10\%<D^*<20\%$, though the portion of reads mapped reduces to 50% or less (Fig. 2g). Overall, except for the most similar genomes with $D^*<1\%$, bowtie2 always maps fewer reads. The mean distances from krepp more closely capture genome-wide distance $D^*$ than bowtie2 when $D^*>8\%$, whereas, for the less novel genomes, bowtie2 has a slight advantage (Additional file 1: Fig. S5). The range of $D^*$ where bowtie2 underestimates ANI coincides with the range where the efficiency of its read mapping degrades, showing that the underestimation may simply be due to unmapped reads (Fig. 2e, g). Computing ANI and $D^*$ using alternative methods such as skani [49] and orthoANI [50] shows similar trends, except for very high distances, $D^*>15\%$ (Additional file 1: Fig. S6). At these levels, krepp is still more accurate in computing mean distance compared to alignment but appears to underestimate the distance computed using orthoANI and skani.

#### Scaling to ultra-large reference datasets

Beyond accuracy, krepp also enjoys better scalability than alignment with bowtie2. As the number of references increases from 2000 to 15,953 (WoL-v2), bowtie2’s running time increases 10$\times$ while krepp’s increases less than 5$\times$, leading to >8$\times$ improvement over bowtie2 in WoL-v2 (Fig. 2h). Similarly, krepp can map 10M short reads against 50,752 (a RefSeq subset) genomes in $\approx$10 minutes, whereas bowtie2 can only map against 5000 (a WoL-v1 subset) genomes using the same amount of time. Both methods scale similarly with the number of reads (Additional file 1: Fig. S7a). The total time needed to build the library is also significantly shorter for krepp than bowtie2 (e.g., 0.5$\times$ for WoLv1), and the latency of krepp can be reduced 16$\times$ by simply dividing into 16 parallel batches (Additional file 1: Fig. S7b). Note that bowtie2 solves a more difficult problem (alignment) than krepp; these comparisons are to clarify that if the only goal is to compute the distance of a read to reference genomes, then using krepp, and hence avoiding the difficult alignment step, is preferable to bowtie2.

The multitree of colors and the LSH index constitute the majority of the memory used by krepp. This memory consumption is comparable to or better than the standard memory-efficient alignment method, bowtie2 [36], which uses the compact FM-index [51] (Fig. 2i). Moreover, krepp requires significantly less memory when building the index due to its distributed memory algorithm, enabled by splitting the LSH index into multiple sets of buckets (i.e., batches). This approach allows creating unified indexes for ultra-large datasets exceeding 100,000 microbial genomes (a RefSeq subset [52] that we will discuss later) on standard machines with 256GB memory, whereas bowtie2 fails due to its higher peak memory usage.

#### krepp accurately places reads on a large phylogenies using distances

**Fig. 3:**
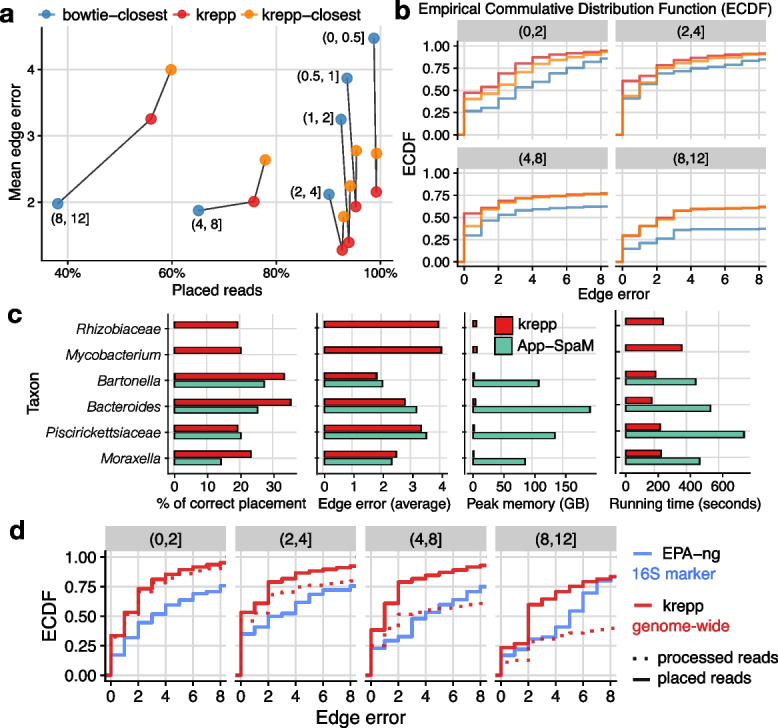
**a**, **b** Placement error and placement portion for 110 query genomes, binned based on novelty. Here, we measured novelty as the path length to the closest leaf on the backbone tree, given as the label next to each triplet in (**a**) and titles of panels in (**b**) and (**d**) (scaled by 100$\times$ for readability). In (**a**), unplaced reads are ignored in computing mean error; in (**b**), shown empirical cumulative distribution function (ECDF) of edge errors treats unplaced reads as infinity error. **c** Comparison of krepp and App-SpaM on six taxa pruned from the WoL-v1 trees. Measurements were performed on 2.10 GHz Intel Xeon Silver 4110 CPUs using 16 threads and 188GB DRAM. App-SpaM ran out of memory for *Mycobacterium* and *Rhizobiaceae*. For comparability with App-Spam, we forced krepp to place every read (i.e., including root placements and no filtering based on the number of matches). **d** Comparing 16S marker-based placement using the maximum likelihood method EPA-ng to genome-wide read placement using krepp on 100 query genomes. We show the error distribution for placed reads and processed reads separately (dashed versus solid); EPA-ng places all 16S rRNA reads given to it as input. The total number of reads analyzed differs immensely (608 for EPA-ng versus 1M for krepp)

We first used the WoL-v2 dataset to compare krepp placements to placing as the sister to the closest reference using distances from either krepp (krepp-closest) and bowtie2 (bowtie2-closest). We selected 110 query genomes from the WoL-v2 reference tree, ensuring a range of novelty levels, measured as path length on *T* to the closest leaf (Additional file 1: Fig. S4) instead of genome-wide distances as done for distance benchmarking (thus, novelty can go beyond 1). We pruned each query genome from the backbone in a leave-one-out manner, generated synthetic 150bp Illumina reads, and placed reads on the tree. We measured error as the number of edges and the total path length (scaled by 100$\times$ in Fig. 3) between each placement and the original position of the edge of the query before pruning (see Benchmarking).

The mean edge error of krepp is low despite placing most reads (Fig. 3a and b). Even for the most novel query genomes, the mean placement error is 3.2 edges (median: 1 edge) despite placing >50% of the reads on the WoL-v2 tree with 15,952 taxa. As novelty levels decrease, more reads are placed, reaching close to 100% for novelty $\le 0.005$. The placement error of krepp increases substantially at the highest levels of novelty (e.g., $(0.04-0.08]$). At the other end, for the least novel queries that are near identical to some references (e.g., $<0.0002$ novelty), the edge error is substantially higher than the $(0.0002-0.002]$ range (Additional file 1: Fig. S2c); the error in path length also increases for these near-identical queries, though to a lesser degree than the edge error (Additional file 1: Fig. S2d). The increase in error in these queries is likely because placing among a large number of highly similar genomes (e.g., *E. coli*) is challenging. Simply using the closest krepp hit increases the error, both in terms of edge error (Fig. 3a) and path length error (Additional file 1: Fig. S2b). Similarly, placing each query as the sister to the lowest common ancestor of all references that are indistinguishable from the minimum krepp distance performs worse than krepp (Additional file 1: Fig. S8a). Compared to bowtie2-closest, krepp-closest places slightly more reads and has a substantially lower, except for most novel queries, where it maps far more reads but has worse accuracy. If we consider unplaced reads as an error of infinity, krepp retains a median error of 0–2 in all levels of novelty. It also finds the correct edge for 26–61% of reads, depending on the novelty; bowtie2-closest has a median error of 1–4 and does not place 50% of reads across the two most novel bins (Fig. 3b).

We next compared krepp to the only alternative method that can place genome-wide short reads, App-SpaM. Due to the high memory demand of App-SpaM, we restricted reference phylogenies to small clades selected from the WoL-v1 tree. For eight clades (six genera and two families, detailed in Additional file 2: Table S1), we selected 40 or 50 genomes as references and the rest as queries, which were all removed from the tree and used to simulate Illumina reads (leave-all-out). Here, krepp is slightly more accurate and far less memory-intensive than App-SpaM (Fig. 3c). App-SpaM requires 84–189GB of memory for these small clades, and we could not build a library for two of the six clades. krepp requires only 1–6GB and is roughly 2–4x faster, including for index construction time (Fig. 3c). Both methods have similar errors, though on average, krepp has 0.15 fewer edge errors compared to App-SpaM and places 6% more reads on the correct branch. Substantial errors by both methods underscore the difficulty of placing reads on densely sampled groups of very similar genomes (Fig. 3c). Note that App-SpaM was at least as accurate as other *k*-mer-based methods, such as RAPPAS [21] and EPIK [20], and the alignment-based APPLES for marker-based placement [19, 20].

Finally, we ask a broader question: Can the marker-free method krepp be competitive or better than using marker-based placement using aligned reads? To test this, we selected 100 genomes that included the 16S marker gene as queries and removed them from WoL-v1 (10,575 tips) and used the rest as the reference for krepp. We generated 150bp reads from 16S marker genes of query genomes, which, unlike genome-wide reads used for krepp, were *error-free*. We aligned these reads using WITCH [53] and placed them using the maximum likelihood method EPA-ng on the reference tree induced to species that include at least one 16S. The edge error of genome-wide reads using krepp is substantially lower than 16S reads using EPA-ng—2.36 versus 5.5 edges on average (Fig. 3d). Improvements are less pronounced but still present except for the most novel level in terms of path length error (Additional file 1: Fig. S8c). In the most novel level, krepp’s performance degrades more noticeably than EPA-ng, and its placement rate substantially decreases. While EPA-ng places all 16S marker reads, placement rates of krepp depend on the novelty, ranging from close to 100% for the least novel bins to below 50% for the most novel bin (Additional file 1: Fig. S8b). Regardless, since we have far more genome-wide reads than 16S, the total number of genome-wide reads placed remains three orders of magnitude higher for krepp compared to reads from 16S genes for EPA-ng (608 versus 1M).

### krepp improves metagenomic sample differentiation

To apply krepp on real metagenomic data, we first re-analyzed a subset of The Human Microbiome Project (HMP) [54] data, consisting of 210 samples, each with 1M subsampled paired-end short Illumina reads [33]. These samples represent seven body sites from both male ($n=$138) and female ($n=$72) subjects. We compared krepp’s ability to characterize these samples against the OGU approach of Woltka [33] (heavily reliant on bowtie2) and taxonomic profiles estimated by Bracken [55]. For krepp, in addition to placements, we also computed OGUs by assigning a read to all leaves of the tree with statistically indistinguishable distances to the minimum. We computed pairwise distance between samples using weighted UniFrac for phylogenetic profiles (krepp and Woltka) or Bray-Curtis for taxonomic profiles (Bracken). We measured the agreement of these distances and the available body part labels using PERMANOVA pseudo-*F* statistics [56] (see Benchmarking).

**Fig. 4:**
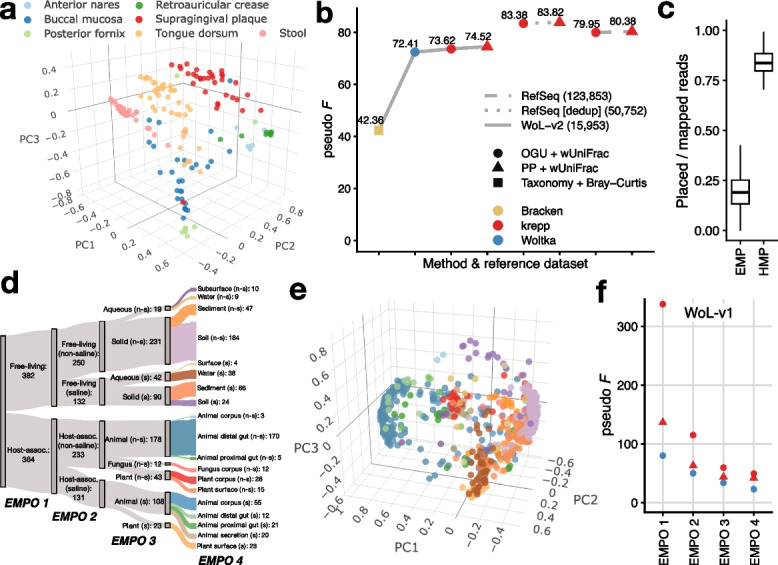
**a**, **e** PCoA of weighted UniFrac distances across samples from Human Microbiome Project (HMP, $n=$ 210) (**a**) and Earth Microbiome Project (EMP, $n=$ 746) (**e**) computed based on krepp’s placements using WoL-v2 and WoL-v1 reference datasets, colored by body sites and microbial environments (EMPO 4), respectively. **b** Separation of body sites in HMP, quantified by pseudo-*F* statistic computed on distance matrices obtained using different methods and reference datasets (WoL-v2, RefSeq before and after deduplication). **c** Ratio of total counts in BIOM tables obtained before rarefication using the OGU approach, comparing reads mapped by krepp (i.e., reads with at least one reference hit) and placed by krepp (i.e., placed below the root, exceeding the $\tau$ threshold) per sample, in EMP and HMP data. **d** Organization of microbial environments according to Earth Microbiome Project Ontology (EMPO) categories, colors of EMPO 4 match (**e**). For EMPO 3 and EMPO 4, n-s: non-saline; s: saline. **f** Separation of microbial environments at different levels in EMP, quantified by pseudo-*F* statistic using WoL-v1 reference dataset, using the same colors and shapes as **b**

Visualized using PCoA, samples separate well in three dimensions using krepp (Fig. 4a). Quantified by pseudo-*F*, krepp resulted in slightly better separation of body sites compared to Woltka (Fig. 4b), noting that OGUs of krepp are better than OGUs of bowtie-2, and placements of krepp are better than its OGUs. Here, the vast majority of reads that are mapped by krepp are also placed (Fig. 4c). Bracken is far less effective than OGUs and placements, a pattern that Zhu et al. [33] attributed to better resolution of phylogeny-based sample distances compared to taxonomy [33].

The real power of krepp for human microbiome analysis is enabling much larger reference data sets. Unlike the alignment-based Woltka, krepp is capable of indexing much larger datasets, enabling us to move beyond WoL references to build indices with 123,853 and 50,752 microbial genomes. The difference between the two sets is that the smaller one removes near-duplicate genomes. While krepp provides small improvements in sample differentiation when WoL-v2 is used, the pseudo-*F* statistic of krepp substantially increases with the larger reference trees (Fig. 4b). These improvements are likely due to better representation of relevant species. Surprisingly, the larger tree with duplicates included does not attain better separation of body sites compared to the deduplicated tree. The reduced separation can be partially explained by the increased ability to capture variation within body sites. For instance, we observed higher distances within the stool category, but anterior nares to supragingival plaque distances decrease by 4% on average (Additional file 1: Fig. S9). Regardless, with both of these trees, krepp’s placements enjoyed higher pseudo-*F* statistics compared to the OGU approach.

Since the human microbiome is relatively well-studied, we next analyzed more novel microbial communities where queries often lack sufficient representation in reference sets [47, 57]. For this, we used the Earth Microbiome Project [58], which includes a wide variety of samples with organisms from non-human hosts and free-living in environments such as soil and water. We analyzed a subset consisting of 746 shotgun metagenomic samples, each with roughly 2M reads on average (in total 14.7B), that were selected and analyzed using Woltka (WoL-v1 reference dataset) by Shaffer et al. [59]. Earth Microbiome Project Ontology (EMPO version 2) organizes these samples into categories at four levels (Fig. 4d) based on host association (EMPO 1), salinity (EMPO 2), host taxon (for host-associated) or phase (for free-living) (EMPO 3), and the specific environment (EMPO 4). We used the same analyses as HMP datasets, focusing on comparing krepp to Woltka at different ontology levels, using the same reference set (WoL-v1) as used by Shaffer et al. [59].

On the EMP data, krepp successfully separates samples by their environmental properties (Fig. 4e). Measured using pseudo-*F* statistic, the distance matrices computed from krepp OGUs reflect EMPO categories remarkably better than alignment-based OGUs at all four levels (Fig. 4f). While improvements are observed in every ontological level (4.2$\times$, 2.3$\times$, 1.8$\times$, and 2.1$\times$ higher at EMPO 1–4, resp.), they are larger at higher levels (Additional file 2: Table S2). Applying the pairwise PERMANOVA test to 19 environment categories at EMPO 4, we observe that 87% of the pairs show significant differences (Additional file 1: Fig. S10). Moreover, cases of insignificant differentiation are highly overrepresented among pairs that include surface (non-saline) and animal corpus (non-saline), which included only four and three samples, respectively. Conversely, soil categories are highly distinguished from all other environments, including between saline and non-saline categories.

While krepp’s placements lead to substantially higher pseudo-*F* statistics than alignment-based OGUs from Woltka, they are not better than krepp’s OGUs (Fig. 4f). The cause of this pattern, which is in contrast to HMP results (Fig. 4b), is not fully clear but may be related to the treatment of ambiguous read mappings during placement. When there are numerous reference hits in divergent clades with distances that are high (e.g., >15%) but indistinguishable, the OGU approach still counts each hit with a weight reciprocal to the number of such hits. However, such reads will most often be placed closer to the root, having little impact on the weighted UniFrac distance compared to the OGU approach. Consistent with this explanation, on EMP, many reads that krepp is able to map (finding a match, regardless of the MPL distance) are not placed (not passing the $\tau$ filter) anywhere below the root (Fig. 4c). On the contrary, the proportion of reads that are placed is much higher relative to the reads that are mapped in the HMP dataset.

### krepp improves taxonomic assignment on CAMI-II

We next tested if krepp distances can be used for taxonomic binning instead of phylogenetic placement. To benchmark krepp’s binning performance, we used the CAMI-II benchmarks [25], focusing on the taxonomic binning of pooled contigs obtained from the gold-standard assembly on their marine and strain-madness datasets. We used our largest index built from 123,853 genomes in the RefSeq snapshot provided for the challenge.

**Fig. 5:**
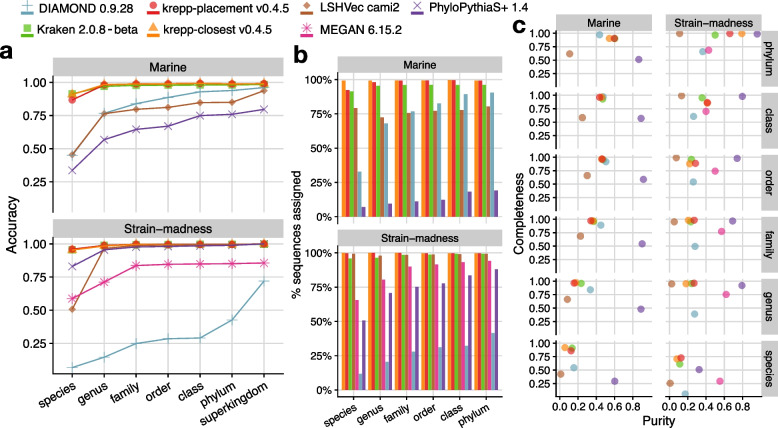
Metrics across ranks in taxonomic binning of contigs of the gold standard assembly of CAMI-II: accuracy of per-contig classifications (**a**), percentage of assigned sequences (**b**), and completeness ($^{TP}\!/_{(TP+ FN)}$) versus purity ($^{TP}\!/{(TP+ FP)}$) where true/false positive/negatives are all defined per taxon without considering their relative abundances (**c**). We compute all metrics as defined by Meyer et al. [25], using AMBER [61]. Each contig is weighted by the number of base pairs it contains in metric calculations, see Additional file 1: Fig. S11 for the unweighted version (contigs are counted equally)

Both versions of krepp performed extremely well according to multiple metrics (e.g., accuracy, completeness, and purity) in both datasets across all taxonomic ranks (Fig. 5 and Additional file 1: Fig. S11). Overall, the krepp-placement exhibits the best binning performance, except at the species level for the marine dataset, where it is slightly behind the previously leading method Kraken2 [60]. No method other than Kraken2 is competitive with krepp. krepp-placement has 2% and 1% higher accuracy (averaged across all ranks), and achieves 29% and 36% less error according to weighted UniFrac (rank invariant) than Kraken2 in marine and strain-madness datasets, respectively (Fig. 5 and Additional file 1: Fig. S11). We also note that krepp’s improvements in accuracy are slightly more pronounced when each contig is treated equally without weighting by the length in metric calculations (Additional file 1: Fig. S11). krepp-closest performs similarly to krepp-placement in terms of accuracy, with differences only at the species level. For the strain-madness dataset, where many closely related strains can be present, krepp-placement does better than krepp-closest, while the opposite is true on the marine dataset. Similar to the trends observed in our previous experiments, krepp-placement and krepp-closest have the highest percentage of assigned sequences, with a considerable increase over the second-best method, Kraken2 (Fig. 5b). Beyond accuracy, which is defined over contigs, we examine purity and completeness, which are defined over taxonomic groups, and weight each taxon equally. krepp-placement achieves noticeably higher purity than Kraken2 consistently in the strain-madness dataset (Fig. 5c) with comparable completeness. Although Kraken2 has marginally higher purity in the marine dataset, krepp-placement compensates by having a commensurate increase in completeness, except at the species level. As expected, krepp-placement creates purer binning than the more aggressive krepp-closest under most conditions. It is possible to further improve the purity at little to no expense in completeness by either relaxing the significance level $\alpha$ or by decreasing the HD threshold $\delta$ (Additional file 1: Fig. S12a). The defaults, on other hand, achieve the best accuracy (Additional file 1: Fig. S12c) and weighted UniFrac (Additional file 1: Fig. S12b) among the alternative configurations tested.

## Discussion

Our method, krepp, provides a practical tool for performing new analyses of modern microbiome data with manageable computational requirements, enabling us to go beyond marker genes while maintaining high accuracy for phylogenetic placement. As the number of references increases, the running time scalability of krepp (Fig. 2h) makes it more attractive than alignment, allowing utilization of larger reference datasets (e.g., a RefSeq snapshot with >120,000 genomes) to increase resolution. Scalability is achieved through LSH-based *k*-mer hashing, which limits the number of *k*-mers comparisons, *k*-mer coloring using a height-bounded multitree, and the fast optimization of a convex single variable likelihood function. To further reduce memory usage, krepp can adopt more aggressive *k*-mer subsampling (which may impact sensitivity), either through minimizers or unbiased sketching methods (e.g., FracMinHash [28, 29]), both of which are currently supported by our software. The default minimization parameters empirically result in retaining approximately 25% of *k*-mers on average across microbial genomes. The trade-off between the accuracy of krepp and the subsampling rate will likely be specific to datasets and remains to be benchmarked systematically. Future work can also incorporate tree-based methods (e.g., KRANK [39]) for *k*-mer subsampling.

Our method has several parameters (e.g., *k*, $\delta$, *h*, *w*, $\tau$ and $\alpha$), which we kept at default values during experiments. We decided on these defaults with a focus on short reads. Future work should explore the alternatives for long reads, contigs, and fragmentary ancient and sedimentary DNA reads. We ignore the impact of sequencing error. For queries with non-random errors and higher error rates, incorporating error models into our likelihood formulation could improve accuracy. Furthermore, systematic testing of krepp with eukaryotic species is needed to assess the impact of large genome sizes and repetitive genomes on accuracy and scalability. The software provides an option for removal of low complexity regions via symmetric DUST algorithm [62]; but the effect of non-random *k*-mer removal on MLP estimates is unknown.

Beyond *k*-mer selection and matching, alternative placement algorithms can also be explored. Our current placement algorithm is branch length agnostic, enabling it to be applied with any binary or multifurcating tree, regardless of how it is computed. Moreover, while its placements are interpretable (sister to the largest clade with statistically indistinguishable distances), the algorithm lacks formal guarantees for trees with high degrees of deviation from ultrametricity. When the backbone tree has branch lengths in interpretable units, we can take advantage of such lengths in the placement algorithm, a topic that we will explore in the future. Such methods could, in particular, address cases with very high levels of deviation from ultrametricity, perhaps using methods such as least-squares error placement [23, 63]. Conversely, our current algorithm needs a rooted tree, a restriction that should ideally be relaxed since accurately rooting trees might not always be trivial. Finally, krepp can provide multiple placements per read, with associated probabilities. Future work should incorporate these measures of uncertainty in the downstream applications, including sample differentiation.

The framework designed here is general and can easily be adopted in other applications. The accurate read-genome distances can also be used for homology mapping (e.g., [35, 64]), taxonomic abundance profiling (e.g., [27, 55]), contamination detection (e.g., [65, 66]), contig-to-contig binning, computing rates of evolution across the genome to detect abnormally fast or slow regions (ultraconserved elements), and perhaps detecting horizontal transfer. Out of practical necessity, we used a species tree in our analyses, and this tree was sufficient for downstream applications such as sample comparison. We note that our placement errors have a long tail of high values, some of which correspond to low distances; a likely explanation is horizontal transfers from genomes that on the species tree are far from the query on the species tree, leading to gene tree discordance [67]. Similarly, we observed highly distant placements (>0.5 tree path length) among statistically indistinguishable placements for 2% of the reads in our WoL-v2 placement benchmarking (Additional file 1: Fig. S13). This observation can be used in the future to design methods that detect HGT or contamination across the genome.

## Conclusions

We introduced the first scalable genome-wide phylogenetic read placement method and, in doing so, developed a novel approach to accurately estimate the distance of a read to a diverse set of genomes. krepp scales well to modern reference sets and requires only a fraction of the computational cost of alignment-based methods for distance estimation. When integrated into widely used metagenomic pipelines based on read alignment to create operational genomic units, krepp demonstrated superior performance in differentiating real metagenomic samples across diverse environments, including both host-associated (human and non-human) and free-living microbial communities.

## Methods

### Locality-sensitive hashing (LSH) index of *k*-mers

Given a query *k*-mer, we seek reference *k*-mers within some Hamming distance (HD) threshold, denoted by $$\delta$$. Expanding on the CONSULT family of methods (CONSULT*) [27, 38, 39], we use LSH with some changes. We use the bit-sampling [68] to partition reference *k*-mers (default: $$k = 29$$) into subsets. The LSH of a *k*-mer *x*, denoted by $$\textrm{LSH}(x)$$, is computed by sampling $$h \ll k$$ (default: 14) random but fixed positions of *x*, providing $$[0,2^{2h})$$ buckets indexed by a 2*h*-bit integer to represent *h* characters from a 4-letter alphabet. For each $$r\in \mathcal {R}$$, we use minimizers by choosing the *k*-mer whose encoding has the smallest MurmurHash3 [69] value in a local window (default 35). We save all surviving reference *k*-mers, denoted by $$\mathcal {M}$$, in the ascending order of their $$\textrm{LSH}(x)$$, breaking ties lexicographically. The result is an array $$\textbf{A}$$ of size $$|\mathcal {M}|$$. We build another ordered offset-index array of size $$2^{2h}$$, denoted by $$\textbf{I}$$ to note the boundaries of LSH partitions; i.e., $$\textbf{I}[i]=\textbf{I}[i-1]+|\{x \,:\, \textrm{LSH}(x)=i, x \in \mathcal {M} \}|$$ and $$\textbf{I}[-1]=0$$. Thus, unlike CONSULT* methods, which limit the size of LSH buckets by removing *k*-mers, krepp uses flexible size partitions and keeps all *k*-mers, which is helpful for distance calculations. To compute the exact HD for *k*-mer matches, $$\textbf{A}$$ needs to store each *k*-mer *x* precisely. Naively, each *k*-mer requires 2*k* bits; however, the position of *x* in $$\textbf{A}$$, together with offset values in $$\textbf{I}$$, already gives the *h* positions used for $$\textrm{LSH}(x)$$; we simply store the remaining $$2(k-h)$$ bits. Thus, $$\textbf{A}$$ requires $$2(k-h)|\mathcal {M}|$$ bits. $$\textbf{I}$$ is much smaller and needs $$\log _2(|\mathcal {M}|)$$ per index (we write $$\log (n)$$ instead of $$\left\lfloor {\log _2(n-1)}\right\rfloor +1$$ when clear by context) and $$2^{2h}\log (|\mathcal {M}|)$$ bits in total. We adopt a left/right *k*-mer encoding [38] that enables computing HD with just four instructions (pop-count, XOR, OR, shift).

Given a query *k*-mer *x*, we only attempt to match it to *k*-mers with the same LSH value (i.e., $\textbf{A}[\textbf{I}[\textrm{LSH}(x)-1]]$ to $\textbf{A}[\textbf{I}[\textrm{LSH}(x)]]$) by calculating the exact HD. The higher the *h* is, the smaller these slices tend to become, but with decreased sensitivity (i.e., more false negatives), especially for higher HD. However, since we explicitly calculate the HD, there are no false positives. Assuming independence of positions, two *k*-mers at HD $= d$ have the same hash with probability ${{k-h \atopwithdelims ()d}}/{{k \atopwithdelims ()d}}$. This probability is sufficiently high for small enough *d* (e.g., $d \le 4$), then drops quickly and diminishes when $d\gg 4$ for appropriate choices of *h* and *k*. For a query sequence of length *L*, the expected number of matches across all $(L-k+1)$ *k*-mers is sufficiently high for several realistic choices of *k* and *h*. The false negatives can be further reduced by using multiple arrays with different LSH functions (randomly sampled *h* positions), a feature that CONSULT* methods use, but we have not tested for krepp. Finally, note that *k*-mer matches at very high HD can be spurious (e.g., not orthologous) and will also have many false negatives as a query *k*-mer and the closest reference match are likely to have different LSH values. Thus, we choose a fixed parameter $\delta$ (default: 4) and only keep *k*-mer matches with HD $\le \delta$.

### Coloring *k*-mers using a multitree based on the reference phylogeny

After finding reference *k*-mers similar to a query *k*-mer using the LSH index, we need to track *which* references include each matched *k*-mer *x* (denoted by $\mathcal {R}(x)\subseteq \mathcal {R}$). It is easy to save one ID for *x*, which is what many methods do (e.g., Kraken [26, 60] tracks the lowest common ancestor (LCA) of $\mathcal {R}(x)$ while CONSULT-II stores a soft-LCA [27]). To calculate distances to all relevant references, we need to record all $\mathcal {R}(x)$ IDs. Keeping pointers from *x* to each $r \in \mathcal {R}(x)$ requires too much memory, necessitating an efficient data structure, which is the colored *k*-mer representation problem [40]. A *color*, denoted by *C*, refers to a subset of references that share at least one *k*-mer ($\mathcal {R}(x)$ for some $x\in \mathcal {M}$). The high-level goal is to compactly represent all colors that are observed in a reference dataset, denoted by $\mathcal {C}=\{\mathcal {R}(x) : x \in \mathcal {M} \}$, and to retrieve them efficiently during query time for matched *k*-mers. For simplicity, we assume all singleton colors ($\{r\}$ for $r\in \mathcal {R}$) are in $\mathcal {C}$. Representing each non-singleton $C\in \mathcal {C}$ as the union of other colors in $\mathcal {C}$ defines a DAG $G=(V,E)$ with colors as nodes and edges representing the partitioning. *G* can be stored in an array of size $|E|+|V|$ (by saving the count and the indexes of children of each node). The trivial bipartite DAG partitioning each non-singleton color into all its constituents will have $|E|=\sum _{C\in \mathcal {C}}|C|-|\mathcal {R}|$. However, we can do better by adding new nodes (i.e., “meta”-colors) for shared patterns across observed colors, which can potentially reduce the size w.r.t. $|E|+|V|$ (Additional file 1: Fig. S1). With meta-colors, we can further reap extra benefits from allowing exactly two children per node. Such a DAG can be stored in an array with 2|*V*| (instead of 3|*V*|) elements, each $\log (|V|)$ bits; the array index is the ID of the color, and for each index, we store the IDs of its children. For each *x*, we keep the index of $\mathcal {R}(x)$ in an array $\textbf{C}$ laid out identically to $\textbf{A}$; this adds $\log (|V|)$ bits to $2(k-h)$ bit *k*-mer encodings, which is manageable (e.g., $|V|\approx 2^{22}$ for our WoL-v2 reference $|\mathcal {R}|=15,953$). Additionally, we restrict the children of each node to be disjoint, making our DAG a binary multitree.

We argue that the phylogeny *T* provides a practical and efficient way to build the multitree. Consider a simple evolutionary model. An ancestral genome evolves down *T* accumulating random substitutions under an infinite *k*-mer assumption (similar to infinite sites) where the probability of any *k*-mer mutating twice is zero. Under this model, each $\mathcal {R}({x})$ becomes a perfect character, meaning that it maps exactly to a single clade of *T*. As a result, all the colors in $\mathcal {C}$ will be a node in *T*; it is easy to see that the optimal solution w.r.t |*E*| is simply *T* after contracting internal nodes without a color associated (i.e., removing branches where no *k*-mer mutated). While this model is reasonable for relatively short time scales, genome evolution across phylogenetic scales is far more complex and does not produce perfect characters. To handle this complexity, we build a multitree instead of a tree.

We start with *T* as a multitree, representing each internal node as a color or meta-color. For simplicity, we describe our algorithm for bifurcating trees, noting that extending to multifurcating trees is straightforward by resolving polytomies arbitrarily as a ladder tree around the multifurcating node. This does not affect the placement algorithm as the distance definition for clades naturally extends to polytomies. We partition each $\mathcal {R}(x)$ into the set of clades $C_1, \ldots , C_n \subseteq \mathcal {R}(x)$ of *T* such that no two clades $C_i$ and $C_j$ are sister to each other (i.e., all clades are maximal); note that each $C_i$ can be a singleton. Note that $n=1$ for a perfect character, and $n=|\mathcal {R}(x)|$ in the worst case. Each $C_i$ is already a node of the multitree and thus can be represented with no additional color. For any set of *n* maximal clades, there can exist at most $n-1$ nodes of *T* that have at least one maximal clade under at least two of their children. For each such node corresponding to $\mathcal {R}(x)$, we define a (potentially new) color as the union of the colors of its children. With this procedure, we obtain $\mathcal {R}(x)$ at the LCA of all clades $C_1, \ldots , C_n$. We implement this idea using Algorithm 1, which builds $\textbf{A}$ and the multitree jointly, moving up the tree *T* in a post-order traversal (implemented with nested task-parallelism). We find all *k*-mers shared between children, and for each such *k*-mer, we create a new color from its existing colors (line 19). The only difficulty is checking if the union of two colors already exists ($\texttt{Parent}$), which we implement using an Abelian group (details in Additional file 3: Supplementary Note). Adapting Algorithm 1 to a multifurcating node is done by building and adding indices of its children one by one in an arbitrary order (equivalent to resolving the node as a random ladder tree). For each child, we find its shared *k*-mers with all *k*-mers of previously added children and create new colors by repeating lines 12–21.

Our empirical analysis shows that our heuristic creates reasonably small multitrees with bounded height (Additional file 1: Fig. S14). Our algorithm adds $\approx$500 nodes per genome for a large and diverse reference dataset WoL-v1 [70] with 10,575 microbial genomes (Additional file 1: Fig. S14a). Our procedure adds $n-1$ auxiliary colors on internal nodes, some of which are expected to be shared with other *k*-mers. This heuristic can be considered effective if a small proportion of the $n-1$ added colors remain unobserved after we process all *k*-mers. We empirically observe this pattern; for 74% of non-singleton colors, none of their $n-1$ added colors remain unobserved, and for 90% of colors, only $^{1}\!/_{3}$ or fewer are unobserved (Additional file 1: Fig. S14b).

In addition to the size, we care about the height of colors, which has a direct impact on the running time. This consideration can conflict with the objective of finding the smallest multitree (e.g., the trivial bipartite graph has height 1). In our algorithm, the height is bounded by the phylogenetic tree *T*, and reference trees tend to be sufficiently balanced. On WoL-v2 [3] reference set with 15,953 prokaryotic genomes, the height of the nodes in the resulting multitree is often short, not exceeding 4 for 99.84% of *k*-mers (Additional file 1: Fig. S14c). The average height of the multitree across all *k*-mers is 0.047, compared to 0.092 when genomes are added one by one in a random order. As a result, the inferred tree leads to more *k*-mers with colors of height zero Additional file 1: Fig. S14d). Moreover, the random addition process adds 47% more multitree nodes.

**Figure:**
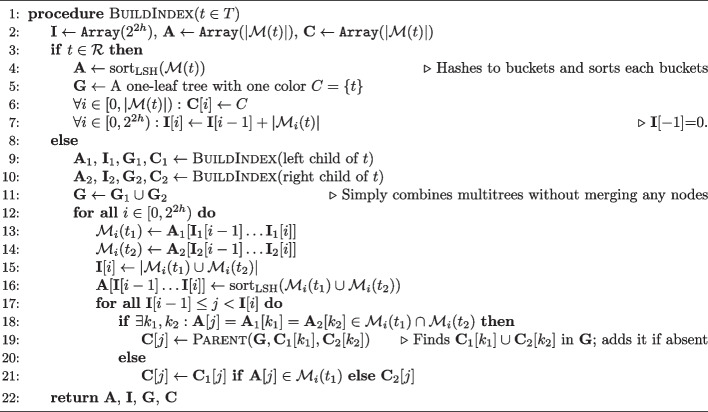
**Algorithm 1** Building the *k*-mer index (LSH index and multitree), given k-mers $\mathcal{M}(r)$ for references $r\ \in\ \mathcal{R}$ and the reference phylogeny T. $\mathcal{M}_i(r)$ is $\{ x \,:\, x \in \mathcal{M}(r), \mathrm{LSH}(x)= i \}$. We implement colors and $C_1 \cup C_2$ ($\texttt{Parent}$) using integer encodings and an Abelian group hashing

### Maximum pseudo-likelihood estimation of the distance

For a query read *q*, we start with all reference genomes with at least one *k*-mer match with HD capped at $\delta$, filter out highly distant genomes when low distant ones also exist (see Additional file 3: Supplementary Note), and let the remaining reference genomes be $\mathcal {R}^\prime$. We compute the distance of *q* to each $r\in \mathcal {R}^\prime$. We make two simplifying assumptions: *i*) The *k*-mer match with the lowest HD is the orthologous one, and therefore, if multiple *k*-mers in *r* match a query *k*-mer, we take the lowest HD and discard others. *ii*) There is no dependency between adjacent *k*-mers, and thus, we ignore positions of matched *k*-mers. Overlapping *k*-mers are clearly not independent, and Blanca et al. [45] have modeled the dependencies for exact matches. However, analyzing the dependency between inexact *k*-mer matches is far more challenging. Thus, we adopt this simplifying assumption, noting that the use of minimizers reduces the number of overlapping *k*-mer matches and reduces dependence. Due to the independence, the likelihood function for distance $D\in [0,1]$ becomes

1 $$\begin{aligned} \mathcal {L}_r(D;k,h,\delta ,u_r,\textbf{v}_r,\rho _r)=\left( P_{\textrm{miss}}(D;k,h,\delta ,\rho _r)\right) ^{u_r}\prod _{d=0}^\delta \left( P_{\textrm{match}}(D;d,k,h,\rho _r)\right) ^{v_{r,d}}. \end{aligned}$$

To compute $P_{\textrm{match}}$, note that if we assume every match is due to homology, observing a match requires three independent events: *i*) The *k*-mer should be indexed despite using minimizers (or any form of random *k*-mer subsampling); we precompute this probability and call it $\rho _r$ (see below). *ii*) Observing *d* mismatches when the underlying distance is *D*; this happens with probability

$$\begin{aligned} P_{\textrm{mutate}}(D;d,k)=D^d(1-D)^{(k-d)}{k \atopwithdelims ()d}\;. \end{aligned}$$

*iii*) The LSH search finds the *k*-mer match at HD $= d$ with probability

$$\begin{aligned} P_{\textrm{collide}}(d, k, h) = \frac{{k-h \atopwithdelims ()d}}{{k \atopwithdelims ()d}}\;. \end{aligned}$$

Therefore,

$$\begin{aligned} P_{\textrm{match}}(D;d,k,h)=\rho _r P_{\textrm{mutate}}(D;d,k) P_{\textrm{collide}}(d,k,h) =\rho _r D^d(1-D)^{(k-d)}{k \atopwithdelims ()d} \frac{{k-h \atopwithdelims ()d}}{{k \atopwithdelims ()d}}\; . \end{aligned}$$

For $\rho _r$, note we exclude *k*-mers only due to the use of minimizers. We precompute $\rho _r$ during the indexing, setting it to the ratio of the number of minimizers to distinct *k*-mers for each *r*.

We can similarly compute the probability of missing a *k*-mer match, which can happen due to either of two disjoint events: *i*) The *k*-mer is not indexed, which happens with probability $1-\rho _r$. *ii*) The *k*-mer is indexed but is not found by our method. An indexed *k*-mer can be missed either because it has HD $>\delta$, and we automatically ignore such matches, or LSH does not find it (a false negative) even when HD $\le \delta$. The probabilities of these two events are respectively $\sum _{d=\delta +1}^k P_{\textrm{mutate}}(D;d,k)$ and $\sum _{d=0}^\delta P_{\textrm{mutate}}(D;d,k)(1-P_{\textrm{collide}}(d,k,h))$. Therefore, we end up with

$$\begin{aligned} P_{\textrm{miss}}(D;k,h,\delta ,\rho _r)=(1-\rho _r) + \rho _r\left( \sum \limits _{d=0}^k P_{\textrm{mutate}}(D;d,k)\left( 1- {1}\{d \le \delta \}P_{\textrm{collide}}(d,k,h)\right) \right) . \end{aligned}$$

For each reference *r*, we maximize its likelihood function to get an estimate for *D*. Equivalently, we maximize the log-likelihood, which considerably simplifies Eq. (1) and $P_{\textrm{match}}$ but does not help with the $P_{\textrm{miss}}$ due to summation. After dropping constant terms (w.r.t *D*), we get:

2 $$\begin{aligned} \hat{D}_r = \underset{D}{\mathrm {arg\ max}} \, u_r \log \left( P_{\textrm{miss}}(D;k,h,\delta ,\rho _r)\right) + \sum \limits _{d=0}^\delta v_{r,d} \left( d\log (D)+(k-d)\log (1-D)\right) . \end{aligned}$$

The log-likelihood function (Fig. 1b) is concave for $D\in (0,0.5)$ provided that *k*, *h*, and $\delta$ follow restrictions given in Additional file 3: Supplementary Note, including for the default values. We use the simple Brent’s method [71] for solving the optimization, which in preliminary analyses was faster than alternatives, such as L-BFGS-B [72]. Brent’s method uses quadratic interpolation to locate the global minimum by numerically approximating the derivative of single-variable convex functions.

### Placement on phylogenies and taxonomies using likelihood-ratio test

When *T* is ultrametric, the distance from *q* to all leaves of its sister clade *C* is the same and is minimum across the tree (Additional file 1: Fig. S2a). Therefore, given true distances, a valid approach is to place *q* as the sister to all references that have the minimum distance to it. This idea remains reasonable when the tree is not fully ultrametric but is sufficiently close to ultrametricity. Since distances calculated from reads have low resolution and high variance, we also need to consider that distances to many reference genomes may be statistically indistinguishable. Although one can consider using the least squares error minimization approach of APPLES [63] in this framework, we opt not to, as it faces a subtle challenge (see Additional file 3: Supplementary Note) related to variable rates of evolution across the genome. We need methods that consider the high variability and noise of distances. We propose a principled likelihood-based test of statistical distinguishability for distances. With such a test, our goal becomes: Find the largest clade *C* of the reference tree *T*, such that the distance of *q* to references in *C* is statistically indistinguishable from the minimum distance of *q* to any node. We base our algorithm on this idea with a particular definition of clade distances (see below). Our formulation needs a root, which is often available and can otherwise be obtained with little effort [73].

#### Distance to a clade

On a bifurcating tree with bounded deviation from ultrametricity, *q* has low mean distances to both left and right children at the correct placement edge or any edge below it; on other nodes, it will have a high distance on one child and a lower distance on the other (Fig. 1b). To capture this intuition, we extend the notion of distance to a clade *C* parent to clades $C_1$ and $C_2$ by first defining $\textbf{v}_C=^{1}\!/\!_{2}(\textbf{v}_{C_1}+\textbf{v}_{C_2})$. We also define $\rho _C=\max (\rho _{C_1},\rho _{C_2})$. Similarly, for multifurcating trees (e.g., a taxonomic tree), we can define $\textbf{v}_C=^{1}\!/\!_{|\textrm{child}(C)|}\sum _{C^\prime \in \textrm{child}(C)}\textbf{v}_{C^\prime }$ and $\rho _C=\max (\{\rho _{C^\prime } : C^\prime \in \textrm{child}(C)\})$ where $\textrm{child}(C)$ denotes the children of *C*. With these definitions, Eqs. (1) and (2) are extended to clades and are used to compute the read-to-clade distances (Fig. 1b).

#### Statistical distinguishability

Small maximum pseudo-likelihood distances $d_\emptyset$ and $d_A$ to two clades $C_\emptyset$ and $C_A$ may differ due to random noise or small changes in $\rho$ (a clade can be just a single reference). We can use likelihood to statistically test for this. Let $C_\emptyset$ be the one with the lower distance, and let $l_\emptyset$ and $l_A$ be the log-likelihood (log of Eq. (1)) computed at $d_\emptyset$ and $d_A$, using $\textbf{v}_\emptyset$ as data. The likelihood ratio test (i.e., $\chi ^2$ test with 1 degree of freedom on $2(l_A - l_\emptyset )$) can be used to test if the higher likelihood clade is statistically distinguishable from the null (default $\alpha =$ 10% significance level).

#### Finding optimal placement

For every internal node of the tree, we compute the distance and use the likelihood ratio test to determine if its distance is statistically tied to the minimum-distance clade (Fig. 1b). Among all clades where we fail to reject the null hypothesis, we choose the largest, breaking ties by choosing the clade with the smallest distance. Alternatively, krepp can report multiplacements, that is, a set of clades (including tips) with distances indistinguishable from the closest, and their likelihood-based weights [74]. For a placement $C_A$, we compute its weight as the pseudo-likelihood values computed at $d_A$, using $\textbf{v}_\emptyset$ as data. These numbers can then be normalized. We do not place reads if $\sum _{i=0}^{\tau } v_{C,i} \le 1$ for the chosen clade *C* (default: $\tau =$ 2). Furthermore, if the chosen clade is the root, we characterize *q* as unplaced.

#### Distal and pendant lengths

As opposed to alignment-based maximum-likelihood placement methods (e.g., [12, 13]), krepp does not infer the distal length, which is the branch length from the attachment location of the placement to the parent of the chosen clade’s root. Instead, it simply uses the midpoint of the edge. Similarly, krepp does not calculate a pendant length, i.e., length of the attached placement edge, and outputs 0 for compatibility with the jplace format [75].

### Benchmarking

#### Genome evolution simulations

We used simulations done by Stepanauskas et al. [46]. Briefly, starting from a genome *X* as the base, they added mutations to it at random using the Jukes-Cantor model with the Gamma model of rate heterogeneity, with the added caveat that gene boundaries were respected (i.e., stop codons were not disturbed) and mutations fell only on the genes and not the $\approx$5% of the genome that constitutes intergenic regions. The genomic nucleotide distance (GND) from the mutated genome, $X^\prime$, to *X* was computed by counting the actual number of mutations added during the simulation, divided by the length (including intergenic regions). Each *X* was mutated at various nucleotide diversity levels ($D \in \{0\%, 0.05\%, 0.1\%, 0.2\%, \dots , 1\%, 2\%, \dots , 19\%\}$) to obtain $X_0$, $\dots$, $X_{30}$. We compare *X* with all $X_i$ ($i\in [0, 30]$) genomes. As *X*, 29 bacterial genomes with high completion levels were used (available at NCBI under bioproject ID GenBank: PRJEB33281) [47]. Rate heterogeneity was modeled by drawing a relative rate multiplier $r_j$ for each gene *j* of *X* from a Gamma distribution with mean 1 and variance $^{1}\!/_{\!\alpha }$ (here, $\alpha =$ 5), keeping rates the same across all levels. A fixed rate was assigned per gene to the base genome *X*, which is used for all $X_i$. Finally, using ART [76], we simulated 150bp error-free reads from 899 (29$\times$31) mutated genomes at 1-fold of read coverage (setting-f 1 -l 150 -s 10), then subsampled to 4 million in total (each query contributing equally) using seqtk [77]. To compute the true Hamming distance, we used the coordinates of simulated reads.

#### Distance and placement evaluation on WoL

For benchmarking of krepp distances with real genomes, we selected 500 query genomes across 36 phyla (267 genera) from RefSeq, with varying distances to the closest reference genome in WoL-v2 (Additional file 1: Fig. S4a), quantified by the average nucleotide identity estimation (ANI) computed using Mash [48] (v2.3 with sketch size of $s=$100,000 and $k=29$). Then, we simulated 150bp short reads at high coverage with default Illumina error profiles using ART (setting-ss HS25 -l 150 -s 10), and subsampled to 33M reads in total. To select queries for phylogenetic placement, we clustered leaves of the WoL-v2 tree using TreeCluster [78] and set the length threshold parameter to the following values: 0.25, 0.1, 0.075, 0.05, 0.04, 0.03, 0.02, 0.01, 0.005, 0.001, 0.0005, and 0.0001. Each of these threshold values results in a clustering of leaves, where some remain unclustered as singletons. For all threshold values except 0.25, we selected all singletons that are not singletons with a larger threshold value. Among these, we sampled 10 leaves at random for each level, resulting in 110 query genomes we used for placement benchmarking with respect to the WoL-v2 dataset (Fig. 3a and b).

On these datasets, we compare krepp (v0.4.5) against distances obtained from alignment and placing on the closest tree leaf using bowtie2 (v2.4.1). For all bowtie2 analyses, to find all alignments with high sensitivity, we ran it with --very-sensitive and --all options.

We evaluated the accuracy of krepp’s placements in three different experiments, comparing krepp placements with placing as sister to the closest reference, comparing krepp and App-SpaM [19] on genome-wide placement of short reads from small clades, and genome-wide placement (krepp) versus marker-based maximum-likelihood placement (EPA-ng [13]). We measure error mainly by the number of edges between the output of the methods and the correct placement before pruning, and also by the total path length between the placement edge and the edge of the query. In the path length calculation, the distal length is included but the pendant length is ignored. We simulate genome-wide 150bp short reads as done in the previous analyses with default Illumina error profiles and attempt to place every read on the backbone after pruning the query genome(s).

For 16S comparison (Fig. 3d), we selected 100 queries from WoL-v1 with varying novelty levels based on the closest neighbor in the backbone tree WoL-v1 (Additional file 1: Fig. S4b). Then, we simulated error-prone short reads using the same ART parameters and subsampled to 1M reads for each of these two query sets. Out of 10,575 genomes in WoL-v1, 7813 genomes included a 16S gene. For EPA-ng, we placed 16S reads after removing all genomes without a 16S gene from the reference tree, whereas genome-wide read placement with krepp is performed without excluding such genomes.

#### Real microbiome analysis

Human microbiome samples analyzed consist of the following seven body sites: stool ($n=$ 78), tongue dorsum ($n=$ 42), supragingival plaque ($n=$ 33), buccal mucosa ($n=$ 28), retroauricular crease ($n=$ 13), posterior fornix ($n=$ 10), and anterior nares ($n=$ 6). We used 1M 100bp paired-end whole-genome sequencing reads subsampled in Zhu et al. [33]. krepp processes paired-end reads separately and estimates distances independently. We built count-based BIOM tables [79] (Biological Observation Matrix) using a separate module available as part of Woltka (woltka classify) for krepp (see Additional file 3: Supplementary Note). For Woltka OGUs and Bracken’s species-level profiles, we obtained UniFrac and Bray-Curtis distance matrices from Zhu et al. [33] (which were also calculated using WoL-v2 reference dataset) and used those for comparison. We used the BIOM tables, rarefied to 100,000 reads per sample after filtering relative abundances below 0.01% (removing two samples), to compute pairwise distances using weighted UniFrac (Woltka and krepp) or Bray-Curtis for taxonomy-based methods.

To test improvements when the dataset size increases, we built two new indices based on the ultra-large phylogenetic tree consisting of 199,330 reference genomes inferred using uDance by augmenting the WoL-v2 tree [3]. Among these genomes, we only retained the ones that also appear in the RefSeq snapshot (as of 2019/01/08) provided by CAMI-II benchmarking [25], resulting in 50,752 genomes, and pruned the original uDance backbone tree down to these genomes. Since Balaban et al. [3] removed near-duplicate genomes from the initially curated set of 656,574 microbial genomes, we further expanded the pruned uDance tree by adding near-duplicates from the intersection of this set and the RefSeq snapshot of CAMI-II. Making near-duplicated genomes sisters (with 0 branch lengths) resulted in the tree consisting of 123,853 references.

Earth microbiome samples consist of 746 microbial communities categorized into four levels (as shown in Fig. 4d). Raw shotgun sequence data for these samples is available through Qiita at qiita.ucsd.edu (study: 13114). The total number of reads was 14,720,788,401 (with a mean of 1,947,709 and a standard deviation of 5,446,907) across all samples. We placed all reads in these samples and estimated distances to find OGUs using krepp, then rarefied the BIOM tables obtained using woltka classify to 6,550 reads per sample (following Shaffer et al. [59]), resulting in 686 samples with sufficiently high sampling depth. Note that only 612 samples survived rarefication at the same sampling depth when the alignment was used, demonstrating the impact of the higher mapping rate of krepp. BIOM tables from outputs of all tools were constructed via woltka classify command, and filtering was done via woltka filter, setting --min-percent 0.01 [33]. For PERMONOVA test and UniFrac computation, we used QIIME2 [80] and its diversity plugin [56], setting sampling depth to 100,000 for human microbiome and 6,550 for Earth’s microbiome, and the number of permutations to 1,000. For Earth’s microbiome, we report pseudo-*F* statistics computed in [59] using bowtie2, Woltka, and the same QIIME2 workflow.

#### CAMI-II taxonomic binning of contigs

Reference genomes (a RefSeq snapshot as of January 8, 2019) and the taxonomy used in the CAMI-II experiments are available at cami-challenge.org/reference-databases/ [81]. Contigs from the gold standard assembly can be found at frl.publisso.de/data/frl:6425521/marine/ and frl.publisso.de/data/frl:6425521/strain/, for marine and strain-madness datasets [82], 2.59 and 1.45 gigabases, respectively. For all tools except krepp (DIAMOND [83], Kraken2 [60], LSHVec [84], MEGAN [85], and PhyloPythiaS+ [86]), we used CAMI-II results submitted to the challenge, available at (github.com/CAMI-challenge/second_challenge_evaluation). For krepp, we used the same set of 123,853 reference genomes as in our HMP experiments. This reference set is a subset of the original RefSeq snapshot, obtained by intersecting it with the 656,574 microbial genomes curated by Balaban et al. [3] for their inferred phylogeny. We selected this intersection because we wanted to use the phylogeny from Balaban et al. [3] for library construction, specifically in the HMP experiment. This step resulted in removing 17,824 genomes that were available to other CAMI-II methods, leaving us with 87% of the original genomes. We computed evaluation metrics (accuracy, percentage of assigned sequences, weighted UniFrac, completeness, and purity) using AMBER (github.com/CAMI-challenge/AMBER) [61], exactly as also done by Meyer et al. [25] for other methods.

## Supplementary information

Additional file 1. Supplementary Figures. This file contains Figs. S1-S14.

Additional file 2. Supplementary Tables. This file contains Table S1 and Table S2.

Additional file 3. Supplementary Note. This file contains additional algorithmic and computational details, as well as the software versions and commands used throughout the experiments.

## Data Availability

The krepp indexes used in our experiments are available at https://registry.opendata.aws/kreppref/, and a tutorial can be found at https://github.com/bo1929/krepp/wiki/. The Web of Life databases [3, 70] can be accessed at biocore.github.io/wol/ (WoL-v1) and ftp.microbio.me/pub/wol2/(WoL-v2). We made simulated genomes, all simulated reads, and auxiliary data, together with distance estimates, placement results, and BIOM tables, available on Dryad [87]. krepp, implemented in C++17 and optimized using OpenMP [88], is freely available at github.com/bo1929/krepp [89] and on Zenodo [90] under GPL-3.0 license, together with a user manual and a tutorial. We used the Boost Math library [91] for numerical optimization, and the parallel-hashmap library [92] for hash maps. Key scripts for reproducibility (experiments, evaluation metrics, and figures) are also provided at github.com/bo1929/shared.krepp and on Dryad [87].
