# Supplementary material for: krepp: a k-mer-based maximum pseudo-likelihood method for estimating read distances and genome-wide phylogenetic placement
Source: Genome Biol. 2026 Feb 21;27:108. doi: 10.1186/s13059-026-03999-y (PMC13032499; doi:10.1186/s13059-026-03999-y)
Supplement: Supplementary file 1 — Additional file 1. Supplementary Figures. This file contains Figs. S1-S14. [file 13059_2026_3999_MOESM1_ESM.pdf]

Supplementary Figures

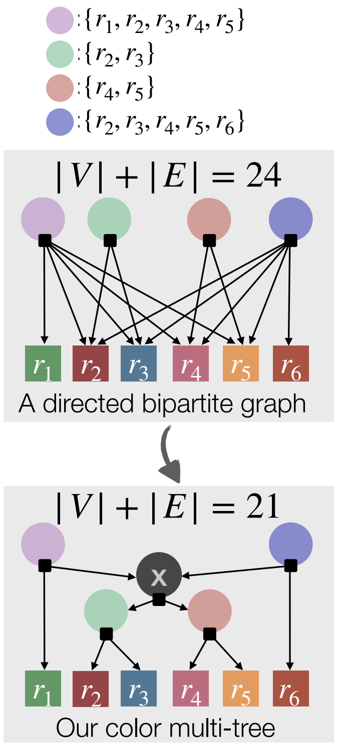

Fig. S1: Top: A trivial multitree with two non-singleton colors (circles) and six references (squares), forming a bipartite graph. Each color is simply the union of its constituents: $\{r_1, r_2, r_3, r_4, r_5\}$ and $\{r_2, r_3, r_4, r_5, r_6\}$. Bottom: The multitree can be made smaller (in terms of total number of edges and vertices, $|E|+|V|$) by adding a meta-color $\{r_2, r_3, r_4, r_5\}$.

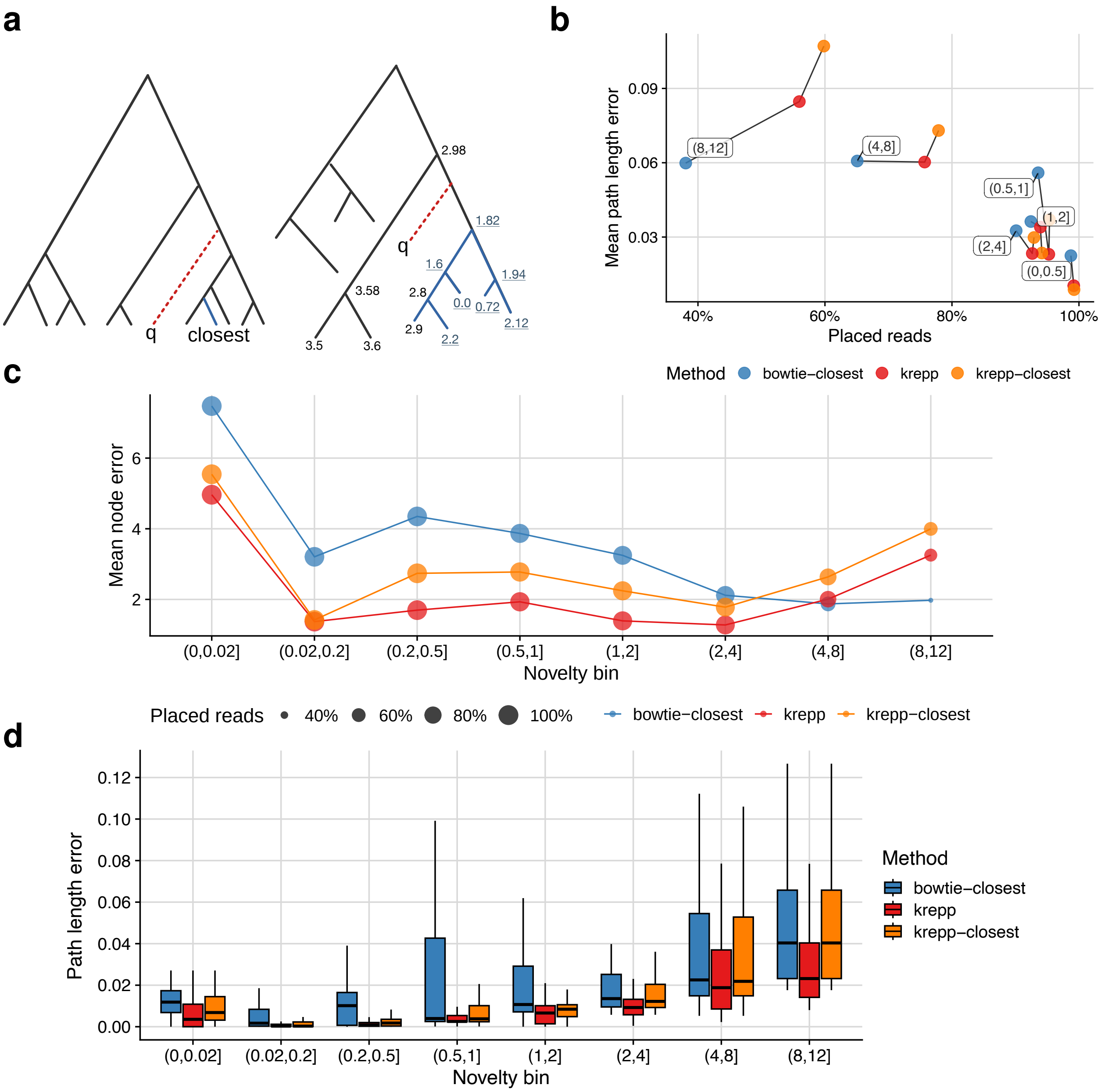

Fig. S2: **a** On an ultrametric tree, the query q has the same minimum distances to its sister clade; thus, the choice of the closest leaf is arbitrary unless the sister clade is a singleton. When the backbone tree is sufficiently close to ultrametricity, distances to the sister clade might be similar and statistically indistinguishable; placing the query as sister to the largest clade of similarly small distances can find the correct placement. Each node is labeled with its χ^2 value according to our likelihood ratio test; all values below 10% significance are indistinguishable (underlined). **b**, **c**, **d** Placement error (y-axis) for 110 query genomes in terms of the edge distance (**c**) and the path length (**b** and **d**). Placement rate is given on the x-axis in **b**. Queries are binned based on novelty (x-axis in **c** and **d**, labels in **b**), measured as the path length to the closest leaf on the WoL-v2 tree (scaled by 100×). Unplaced reads are ignored in computing the mean error in **b** and **c**. The path length error distributions in **c** are only over reads placed by all methods (69% of the all reads).

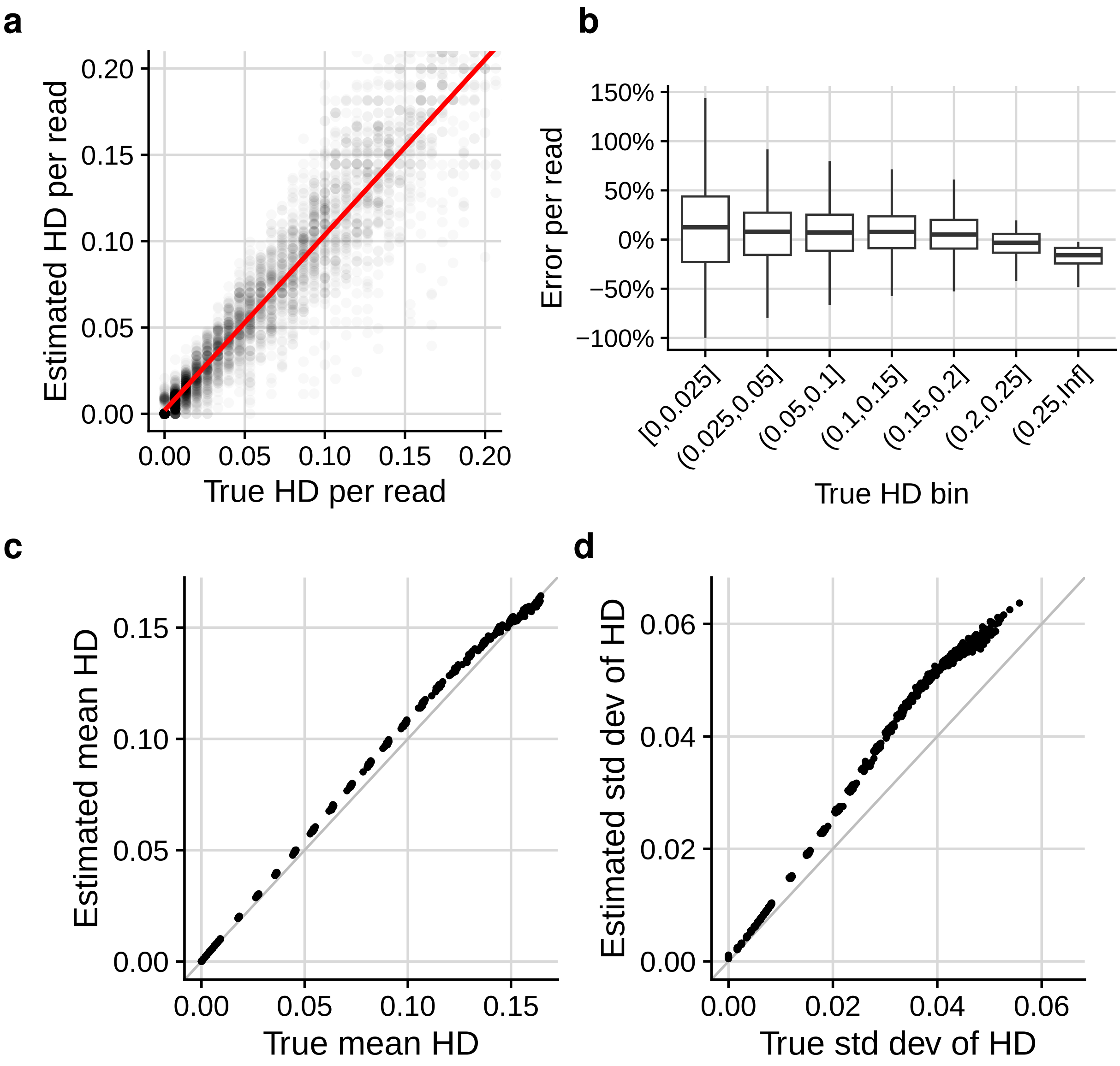

Fig. S3: **a** Comparing true Hamming distance (normalized by the read length) and the estimated HD for individual 150bp short reads. Each data point is a read, and we plot 100,000 randomly subsampled reads in total. The red line is fit using a linear model. **b** Read-level percentage error distributions for each true HD bin, demonstrating small overestimation bias, especially in low Hamming distances. **c**, **d** Each data point is a mutated genome, and the mean (**c**) and standard deviation (**d**) values are computed across all reads that krepp can map to the corresponding base genome.

a

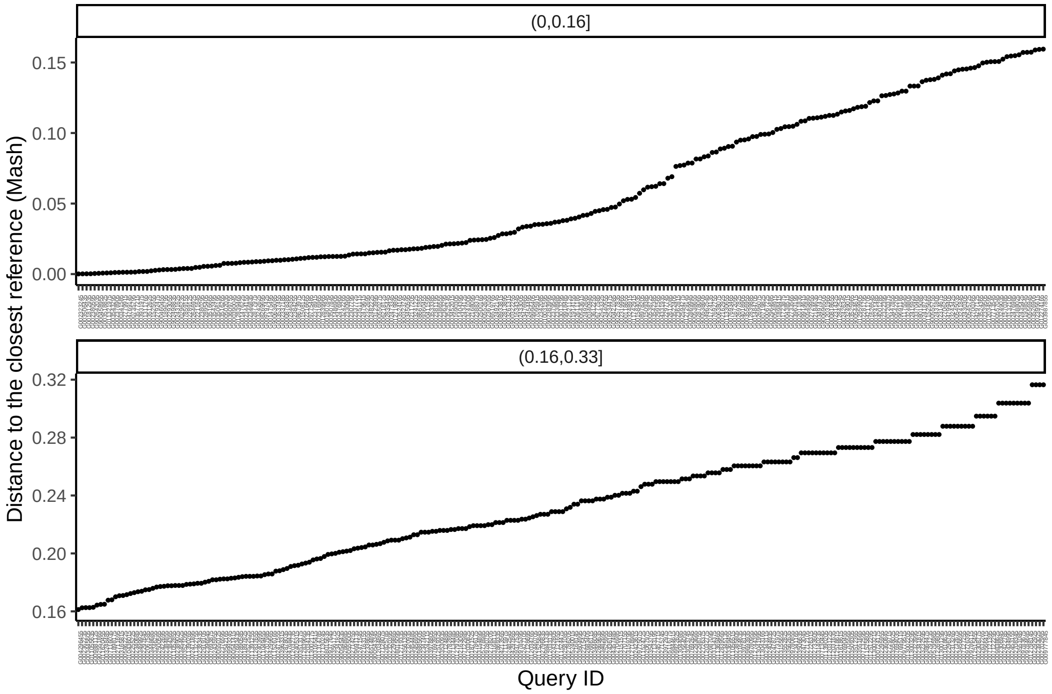

b

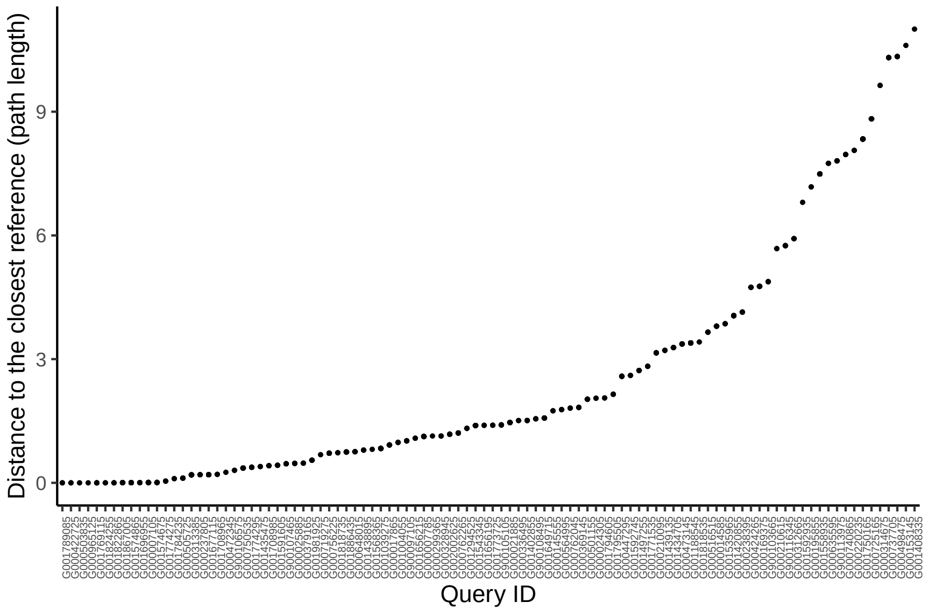

Fig. S4: **a** Distances of 110 queries analyzed for distance benchmarking, measured by 1-ANI to the closest reference in WoL-v2. **b** Path lengths (scaled by 100×) to the closest reference for 100 queries selected from WoL-v1 tree.

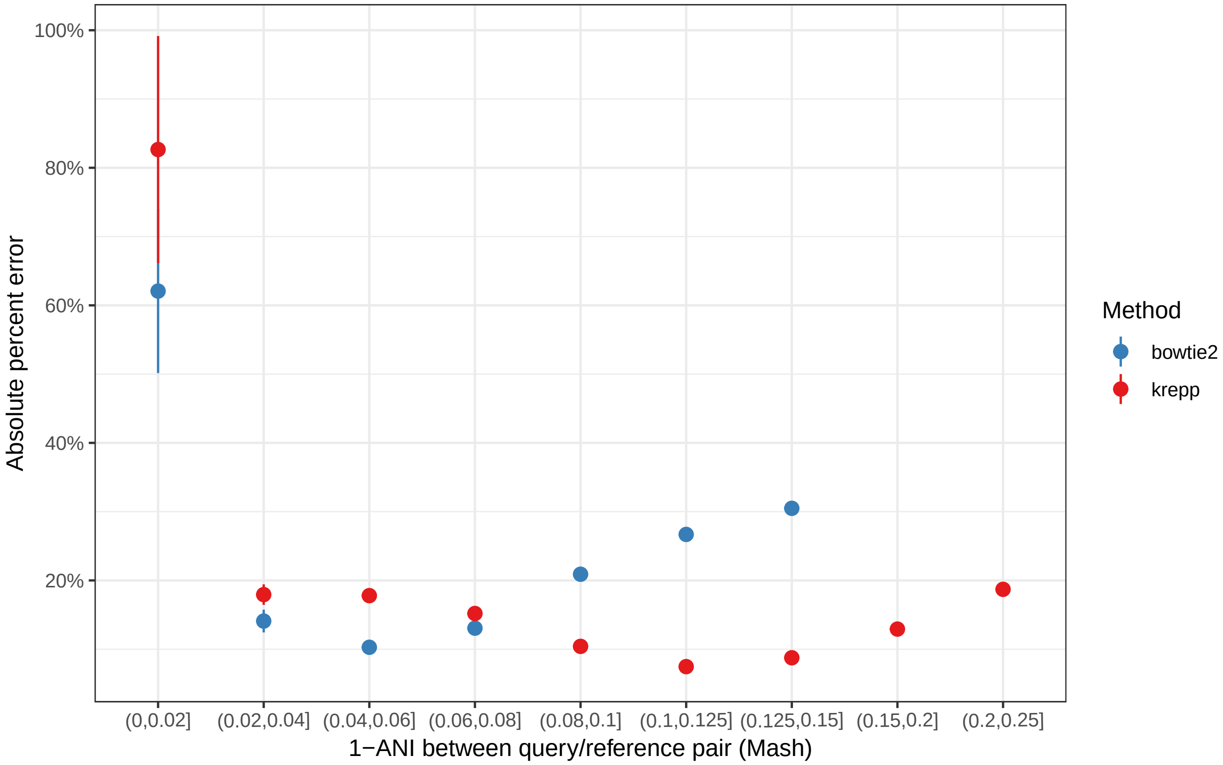

Fig. S5: Change in the relative error ($|\bar{D} - D^*|/D^*$) of krepp and bowtie2 across genome-wide distance bins (measured by Mash) for query/reference pairs with at least 20% reads mapped by each method.

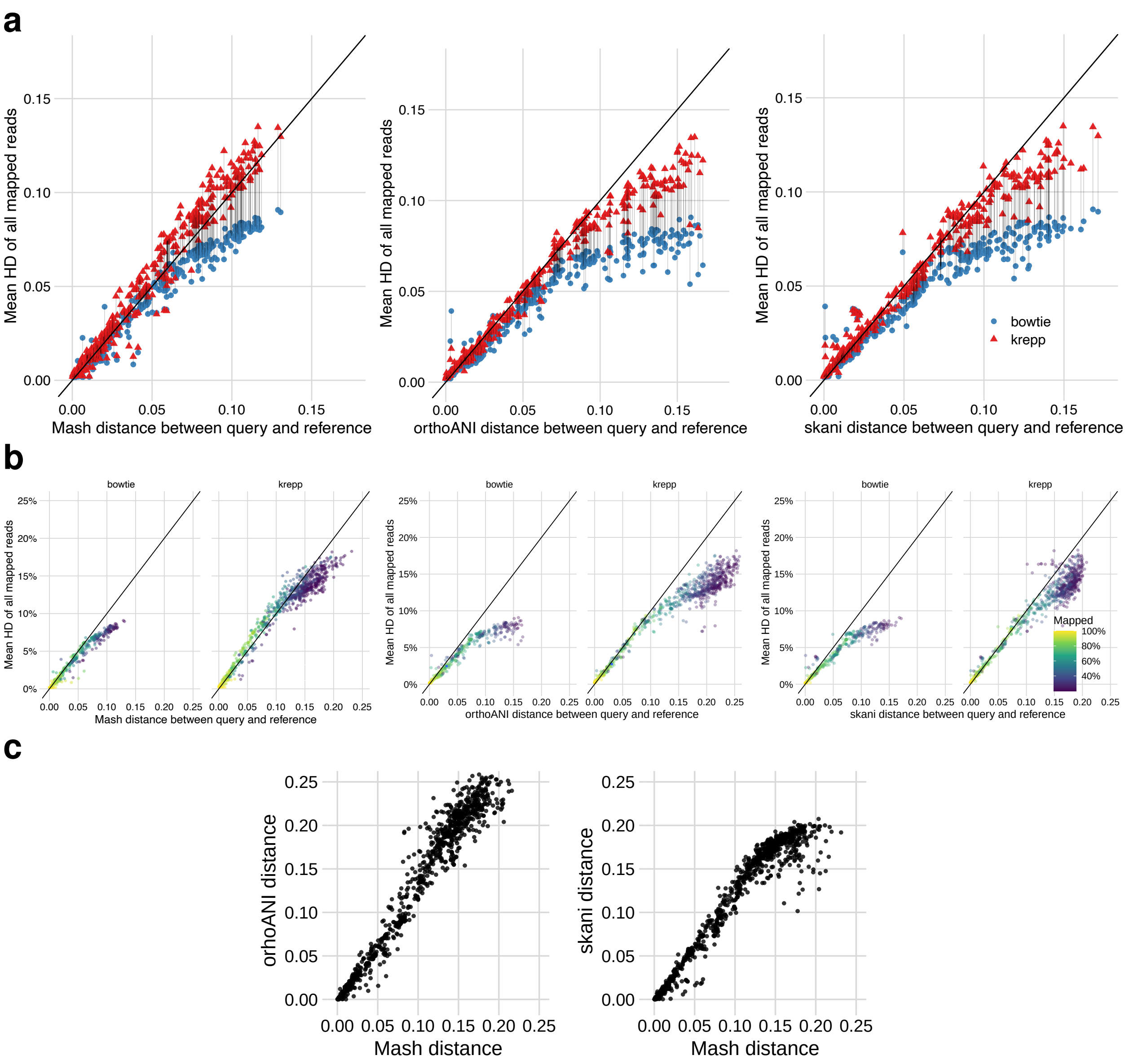

Fig. S6: **a** Mean distance across reads ($\bar{D}$) versus orthoANI [50] and skani [49] genome-wide distance estimates D^* for query/ref genome pairs with ≥20% reads mapped by both methods. **b** $\bar{D}$ versus orthoANI and skani distance estimates D^* from each query to all references with at least 20% reads mapped (colors). **c** Comparing genome-wide ANI estimates of Mash with orthoANI and skani. We set `--min-af` parameter of skani to 0 to output distances regardless of the aligned fraction value. All other parameters of orthoANI and skani are set to defaults.

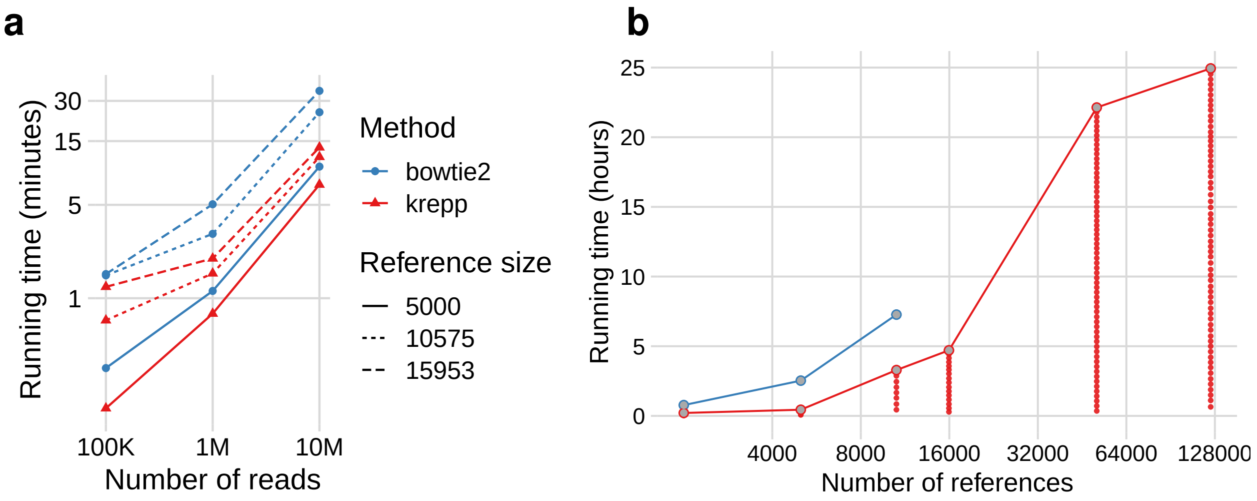

Fig. S7: Scaling. **a** Querying time versus the number of reads. Both methods scale similarly. **b** Indexing time versus the number of references. krepp builds the index in batches (a set of consecutive rows of the LSH index); we show the sum (line), but batches (stacked dots) are run separately in parallel. WoL-v2 reference (15,953 genomes) with bowtie2 had to be built on a more powerful machine, taking 5.5 hours, and cannot be compared.

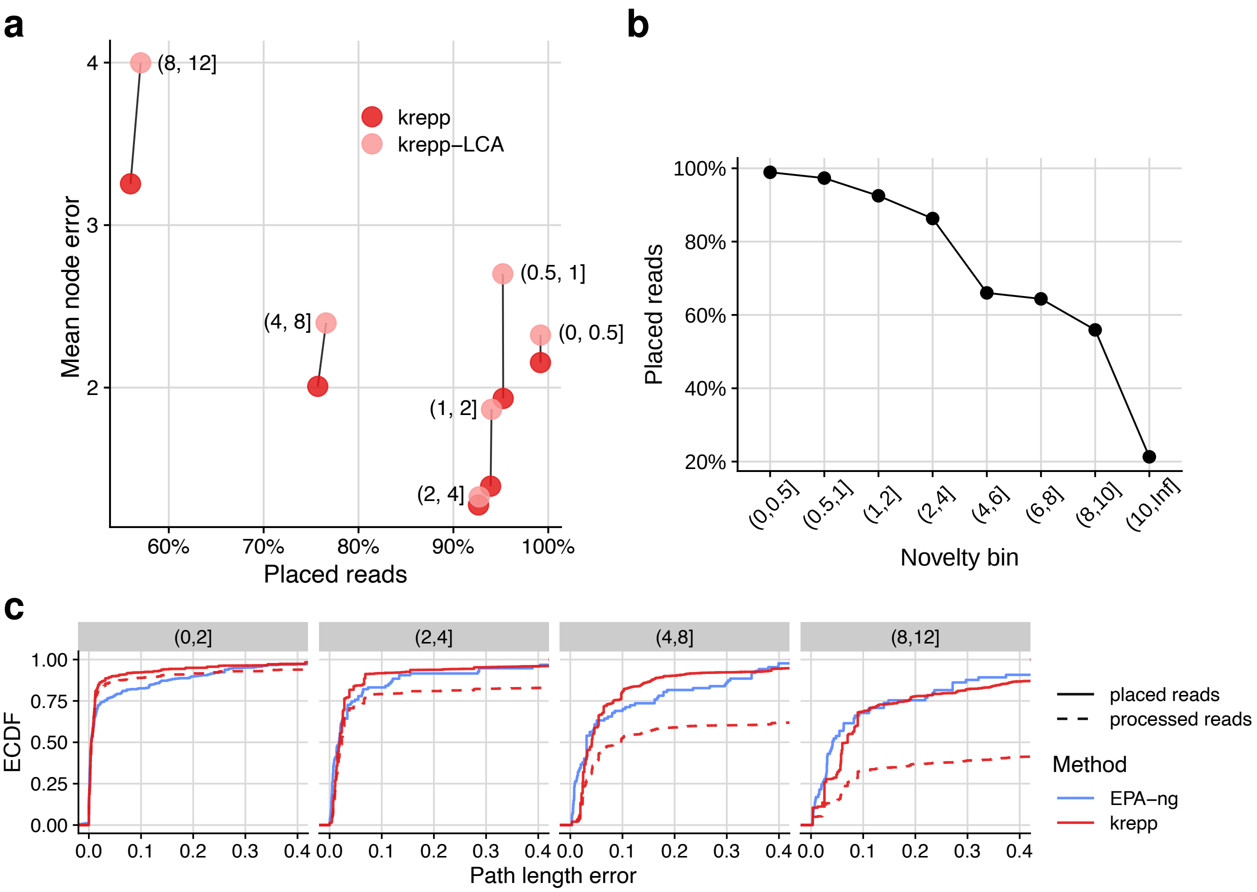

Fig. S8: **a** Using krepp distances and statistical test of distinguishability, we can find the set of leaves that are all tied with the closest distance to the query; the read can then be placed as sister to the lowest common ancestor (LCA) of these leaves. This approach increases the error compared to our default algorithm. **b** Percentage of reads placed for queries selected from WoL-v1 across varying novelty levels. We observed a reduced placement rate compared to using more densely-sampled WoL-v2 as the reference. **c** The empirical cumulative distribution function (ECDF) of path length errors in 100 queries selected from WoL-v1 in a leave-all-out manner. For krepp, we only took the best hit (default). Panels correspond to novelty levels measured as the path length (scaled by 100x in labels) to the closest leaf on the backbone tree. For EPA-ng, we computed the average error of multiplacements weighted by the likelihood weight ratios. We show distribution for placed reads and processed reads (i.e., input read set) separately (dashed versus solid) since EPA-ng places all reads it processes, but is limited to 16S rRNA genes, and the total number of reads analyzed differs immensely (608 versus 1M).

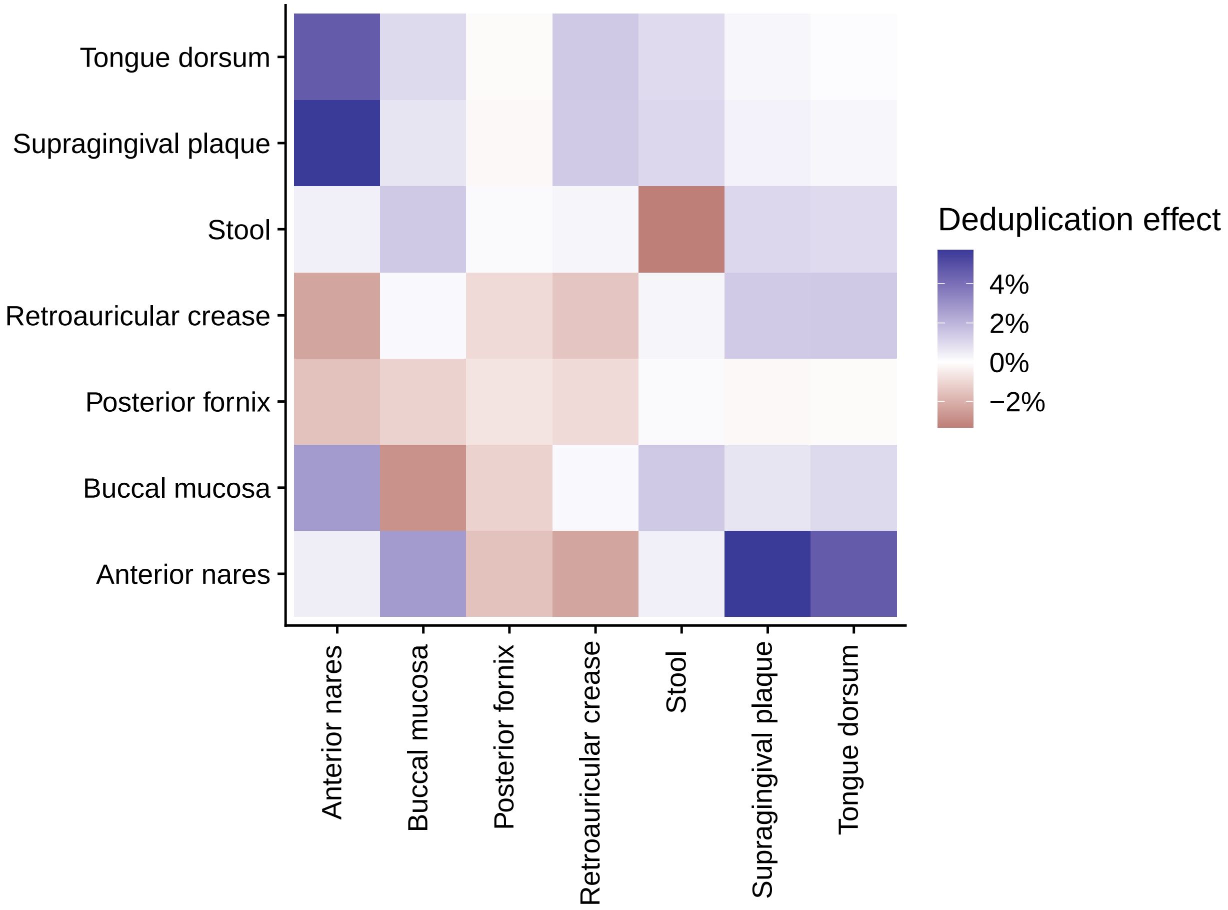

Fig. S9: Effect of deduplication on weighted UniFrac distances across samples from all body sites. We show the average change in distances when krepp placements are computed with respect to the deduplicated RefSeq index, instead of the one with near-duplicates.

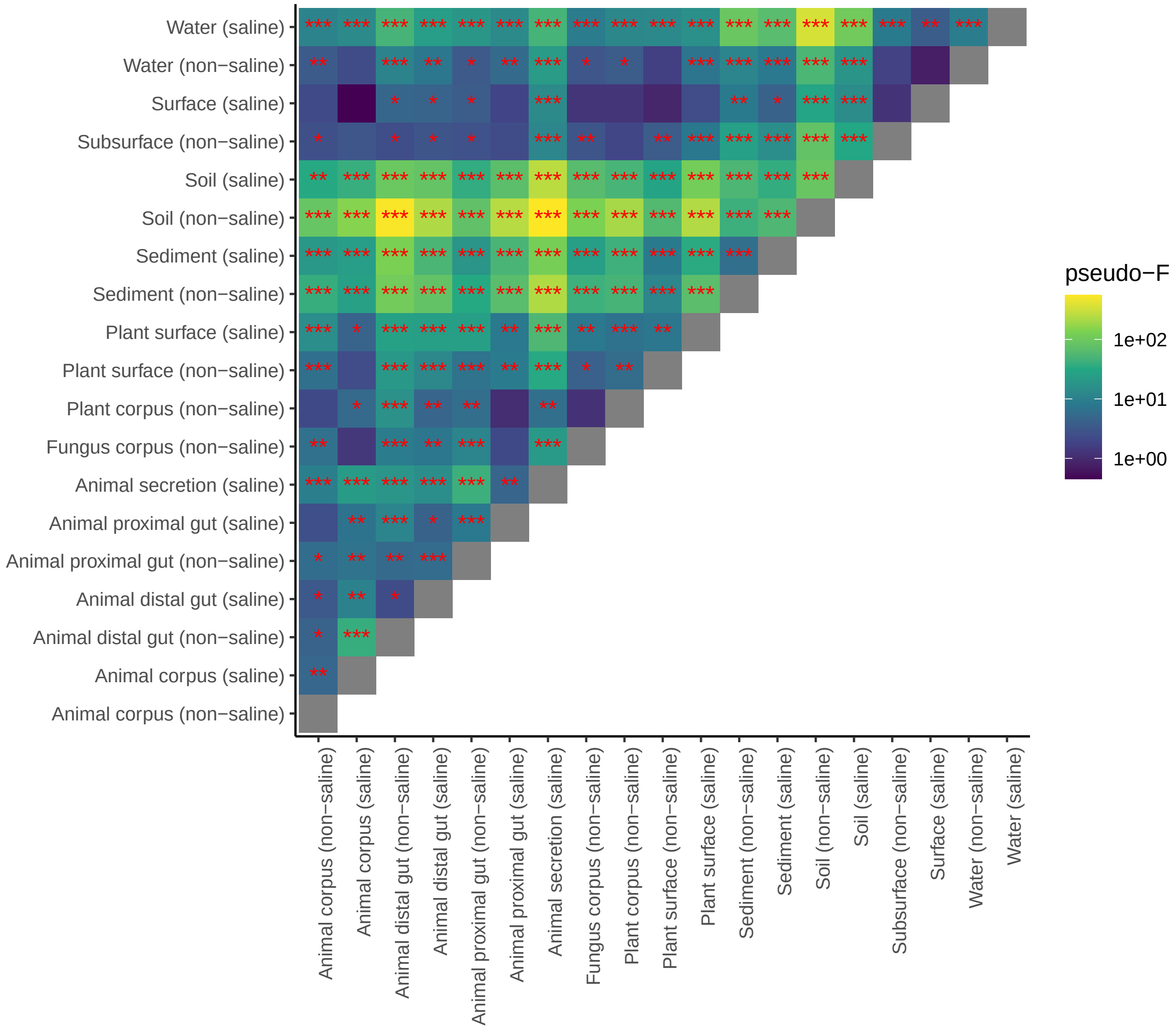

Fig. S10: Pseudo-F and significance of separation based on pairwise PERMANOVA test across different environments at EMPO 4. The number of stars for each environment pair corresponds to different levels of p-value (shown as $*$: $\leq 5\%$, $**$: $\leq 1\%$, $***$: $\leq 0.1\%$).

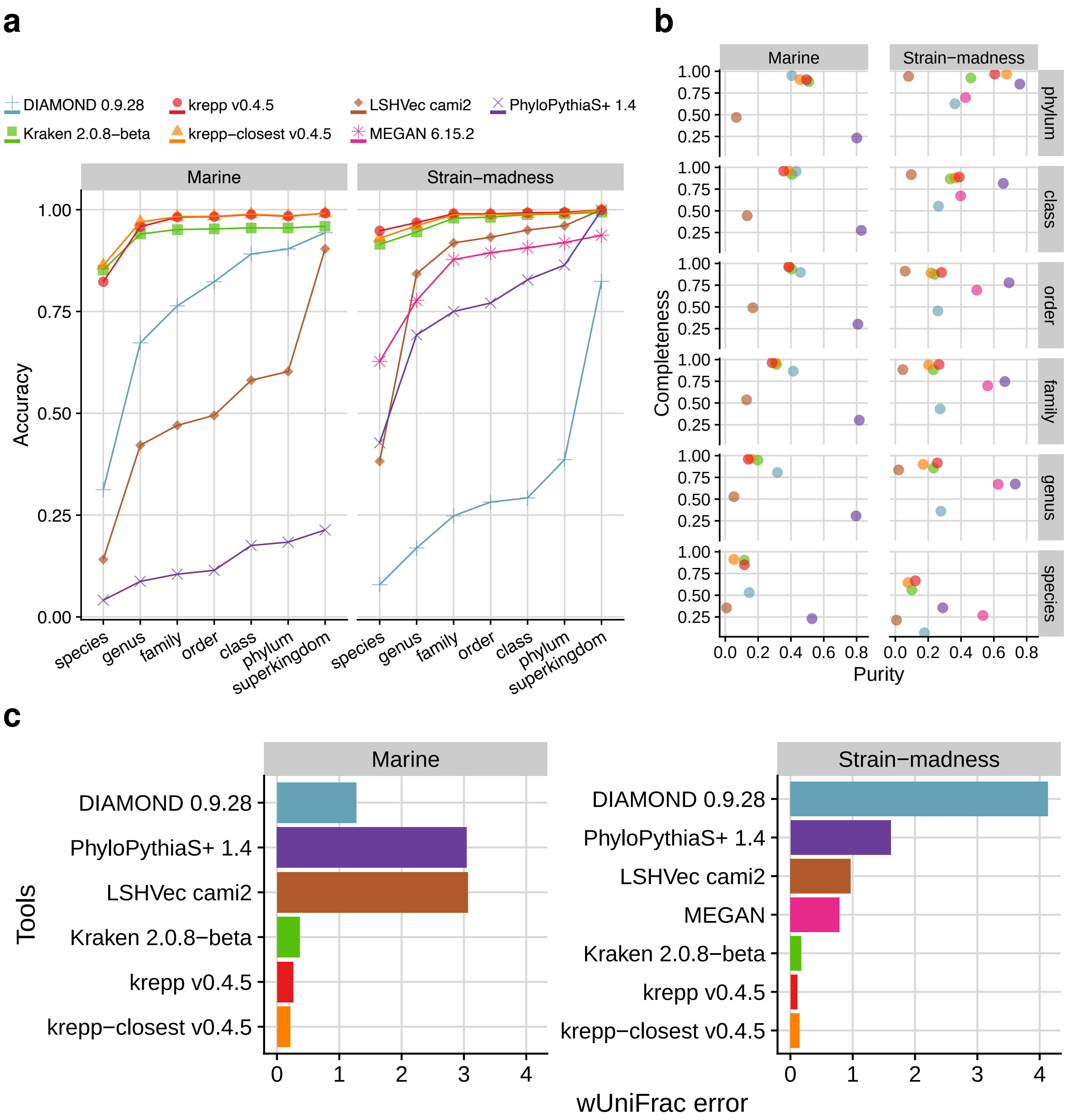

Fig. S11: Accuracy (**a**), completeness versus purity (**b**) across taxonomic ranks, and the rank-invariant weighted UniFrac error (**c**) in taxonomic binning of contigs of the gold standard assembly of CAMI-II. All metrics computed using AMBER [60], reporting metrics that count each contig equally.

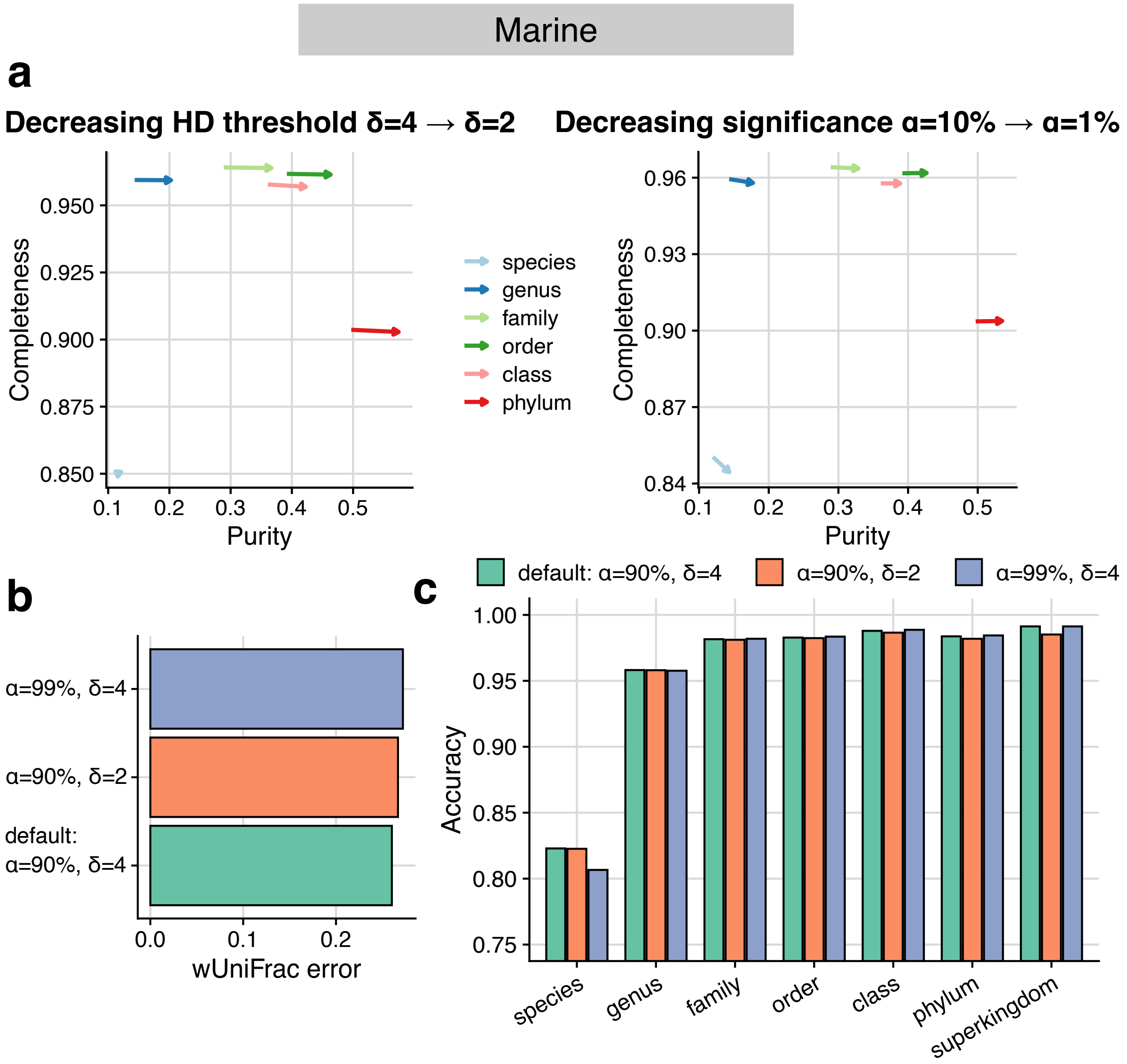

Fig. S12: **a** Impact of hyperparameters δ and α on completeness and purity across varying ranks in the marine dataset . **b**, **c** Comparison of different hyperparameter configurations of krepp in the marine dataset according to weighted UniFrac (**b**) and accuracy (**c**) without weighting by the number of base pairs. The default configuration achieves the best accuracy and the lowest weighted UniFrac error.

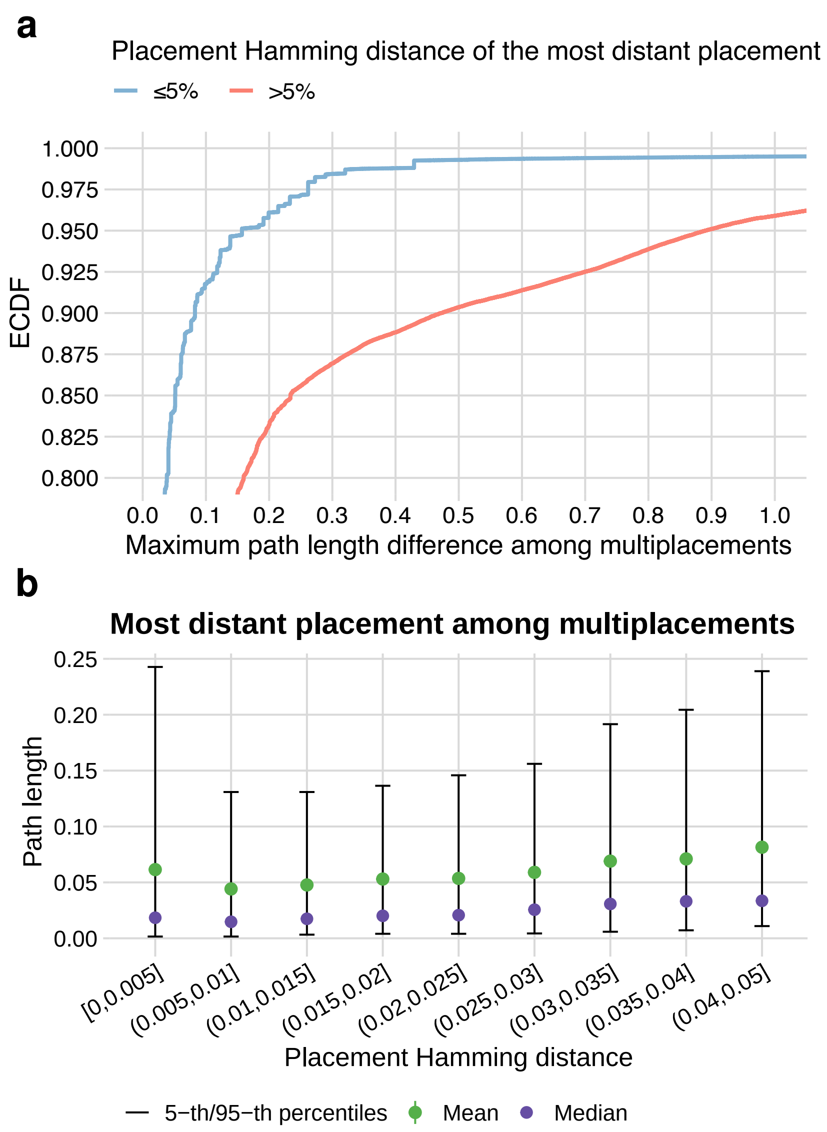

Fig. S13: Multiplacement dispersion for all reads used in the WoLv2 benchmarking. Let $p_1^{(j)} \dots p_k^{(j)}$ be k statistically indistinguishable placements (i.e., multiplacements) of a read j, and let $c^{(j)}$ be the correct placement. We let each $d_i^{(j)}$ be the path length on the backbone tree between $p_i^{(j)}$ and $c^{(j)}$. We focus on the maximum path $m^{(j)} = \max_i d_i^{(j)}$ and the difference between maximum and minimum: $\delta^{(j)} = \max_i d_i^{(j)} - \min_i d_i^{(j)}$. **a)** Empirical cumulative distribution function of $\delta^{(j)}$ across all reads. Query reads are grouped into two by the Hamming distance to the placement that lead to the maximum distance $m^{(j)}$ computed using krepp. For reads that are placed with a fairly high sequence similarity ($\leq$5% HD), we observe a tail of $\approx$9% of reads that with $\delta^{(j)} > 0.1$. **b)** We show mean, median, and the 5–95% percentiles of $m^{(j)}$, binning queries (x-axis) based on the maximum HD, and only showing those with maximum HD up to 5%. Some reads have exceedingly high maximum path length to multiplacements while the HD to that placement is small (e.g., 5% of the reads that have a placement up to 0.5% have $m^{(j)} > 0.24$). These patterns may indicate horizontally transferred regions.

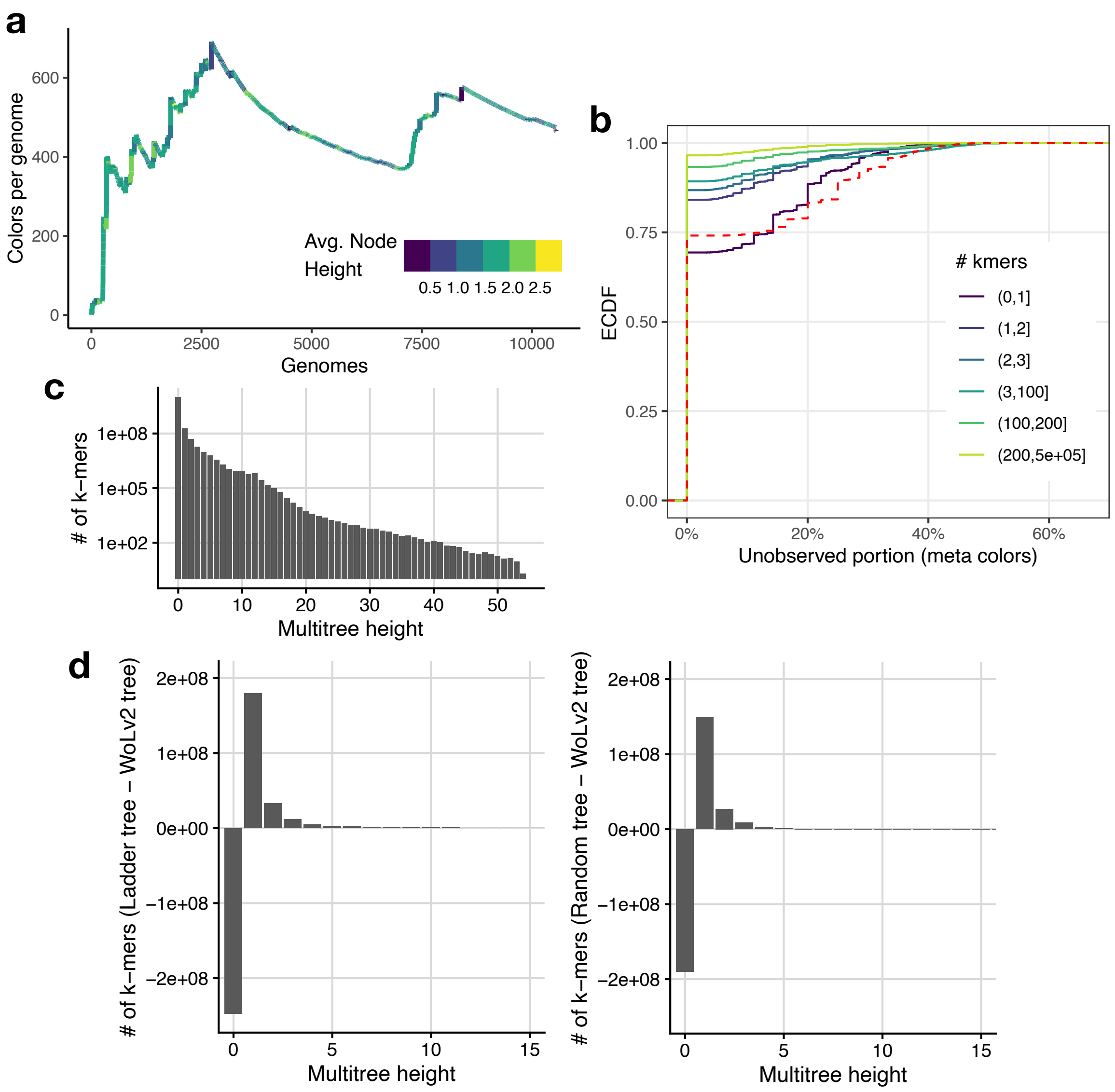

Fig. S14: **a** On WoL-v1 data set (with 10,575 genomes), the number of nodes added per genome while indexing genomes, progressing in postorder along the reference phylogeny (x-axis). **b** For every color in the WoL-v2 reference dataset with 15,953 genomes, we visit the colors below it on the multitree; we then count what percentage of these colors are meta-colors (those that are not observed with any k-mer). We show the empirical cumulative distribution of the portion of colors that are unobserved under each color. We divide this based on how many k-mers are labeled by each color, showing the undivided data in the dotted red line. Colors that encode subsets of references with only a few shared k-mers (i.e., $(0-3]$) are more often represented by meta-colors, while colors that encode reference subsets that share many k-mers (i.e., >100) are almost always also represented by colors observed in the data. **c** The histogram of the number of k-mers with a certain height in the WoL-v2 dataset. Most k-mers belong to colors with height 0, 1, or 2 (97%, 1.8%, and 0.5%, respectively). **d** Comparing the number of k-mers with colors with certain multitree heights when different trees are used. The differences in the number of k-mers between: a random ladder tree and WoL-v2 tree (left), a random dual-birth model tree ($\lambda_A = 10$, $\lambda_B = 1$) [93] and WoL-v2 tree (right) are shown.
