# Supplementary material for: krepp: a k-mer-based maximum pseudo-likelihood method for estimating read distances and genome-wide phylogenetic placement
Source: Genome Biol. 2026 Feb 21;27:108. doi: 10.1186/s13059-026-03999-y (PMC13032499; doi:10.1186/s13059-026-03999-y)
Supplement: Supplementary file 2 — Additional file 2. Supplementary Tables. This file contains Table S1 and Table S2. [file 13059_2026_3999_MOESM2_ESM.pdf]

Supplementary Tables

Taxon	Rank	# of reference	Reference size	# of queries	# of query reads
Piscirickettsiaceae	family	40	119 Mbps	12	600K
Bacteroides	genus	40	171 Mbps	13	350K
Moraxella	genus	40	232 Mbps	14	700K
Bartonella	genus	50	93 Mbps	10	560K
Mycobacterium	genus	40	232 Mbps	21	1.05M
Rhizobiaceae	family	50	235 Mbps	10	475K

Table S1: Details of small reference sets consisting of a single taxon and their corresponding query sets. Query genomes are simply the remaining genomes after excluding randomly selected genomes from WoL-v1.

	EMPO 1	EMPO 2	EMPO 3	EMPO 4	# of samples (after rarefication)
krepp (placement)	136.6	63.0	43.8	41.9	707
krepp (OGU)	337.9	115.0	59.5	49.3	711
Woltka	80.0	50.0	33.7	22.9	612

Table S2: Pseudo-F statistic of weighted UniFrac distances between EMP samples computed at different EMPO levels with respect to the WoL-v1 reference tree.
