# Supplementary material for: krepp: a k-mer-based maximum pseudo-likelihood method for estimating read distances and genome-wide phylogenetic placement
Source: Genome Biol. 2026 Feb 21;27:108. doi: 10.1186/s13059-026-03999-y (PMC13032499; doi:10.1186/s13059-026-03999-y)
Supplement: Supplementary file 3 — Additional file 3. Supplementary Note. This file contains additional algorithmic and computational details, as well as the software versions and commands used throughout the experiments. [file 13059_2026_3999_MOESM3_ESM.pdf]

Supplementary Note

Efficient union of sibling indexes

Algorithm 2 Building the k-mer index (LSH index and multitree).

procedure BuildIndex($t \in T$)
    $\mathbf{I} \leftarrow$ `Array`(2^{2h}), $\mathbf{A} \leftarrow$ `Array`$(|\mathcal{M}(t)|)$, $\mathbf{C} \leftarrow$ `Array`$(|\mathcal{M}(t)|)$
    if $t \in \mathcal{R}$ **then**
        $\mathbf{A} \leftarrow \mathrm{sort}_{\mathrm{LSH}}(\mathcal{M}(t))$ ▷ Hashes to buckets and sorts each buckets
        $\mathbf{G} \leftarrow$ A one-leaf tree with one color $C = \{t\}$
        $\forall i \in [0, |\mathcal{M}(t)|) : \mathbf{C}[i] \leftarrow C$
        $\forall i \in [0, 2^{2h}) : \mathbf{I}[i] \leftarrow \mathbf{I}[i-1] + |\mathcal{M}_i(t)|$ ▷ $\mathbf{I}[-1]$=0.
    else
        $\mathbf{A}_1, \mathbf{I}_1, \mathbf{G}_1, \mathbf{C}_1 \leftarrow$ BuildIndex(left child of t)
        $\mathbf{A}_2, \mathbf{I}_2, \mathbf{G}_2, \mathbf{C}_2 \leftarrow$ BuildIndex(right child of t)
        $\mathbf{G} \leftarrow \mathbf{G}_1 \cup \mathbf{G}_2$ ▷ Simply combines multitrees without merging any nodes
        for all $i \in [0, 2^{2h})$ **do**
            $k \leftarrow \mathbf{I}[i-1]$, $j_1 \leftarrow \mathbf{I}_1[i-1]$, $j_2 \leftarrow \mathbf{I}_2[i-1]$
            while $j_1 < \mathbf{I}_1[i]$ and $j_2 < \mathbf{I}_2[i]$ **do**
                if $\mathbf{A}_1[j_1] < \mathbf{A}_2[j_2]$ **then**
                    $\mathbf{A}[k] \leftarrow \mathbf{A}_1[j_1]$, $\mathbf{C}[k] \leftarrow \mathbf{C}_1[j_1]$, j_1++
                else if $\mathbf{A}_2[j_2] < \mathbf{A}_1[j_1]$ **then**
                    $\mathbf{A}[k] \leftarrow \mathbf{A}_2[j_2]$, $\mathbf{C}[k] \leftarrow \mathbf{C}_2[j_2]$, j_2++
                else
                    $\mathbf{A}[k] \leftarrow \mathbf{A}_1[j_1]$, $\mathbf{C}[k] \leftarrow$ Parent$(\mathbf{G}, \mathbf{C}_1[j_1], \mathbf{C}_2[j_2])$
                    j_1++, j_2++
                k++
            while $j_1 < \mathbf{I}_1[i]$ **do**
                $\mathbf{A}[k] \leftarrow \mathbf{A}_1[j_1]$, $\mathbf{C}[k] \leftarrow \mathbf{C}_1[j_1]$, j_1++, k++
            while $j_2 < \mathbf{I}_2[i]$ **do**
                $\mathbf{A}[k] \leftarrow \mathbf{A}_2[j_2]$, $\mathbf{C}[k] \leftarrow \mathbf{C}_2[j_2]$, j_2++, k++
            $\mathbf{I}[i] \leftarrow k$
    return $\mathbf{A}$, $\mathbf{I}$, $\mathbf{G}$, $\mathbf{C}$

Filtering high distance genomes and placements

We perform the following forms of filtering before computing distances.

- When a read q comes from a region that is shared across many references, the number of matches can be excessively high, especially for high δ (e.g., > 2). For the sake of running time, we ignore references with only high HD matches if low HD matches exist for other references. Specifically, let $d_{\min}$ be the minimum HD across all k-mers for all $r \in \mathcal{R}$. We ignore a reference if the minimum HD across all its matches to q is $2(d_{\min} + 1)$ or higher.
- Optionally, krepp can only report matching references with distances statistically indistinguishable from the MPL distance of the closest reference, using the same likelihood ratio test as the placement. This option is disabled by default.

After computing distances and choosing a candidate clade C (the largest one that we fail to reject the null hypothesis for), we avoid placing a read if it has too few k-mer matches ($\sum_{i=0}^{\tau} v_{C,i} \leq 1$, where $v_{C,i}$ is the average number of matches at HD=i across the children of C, default: τ=2).

Computing Parent and Abelian group hashing

The encodings of colors in $\mathbf{C}$ play an important role in the efficient computation of $\textsc{Parent}(\mathbf{G}, C_1, C_2)$. A desired encoding should enable fast computation of $C = C_1 \cup C_2$, and we also need to query if C was already added to the multitree, G. We do this by using an Abelian group hashing scheme for sets, i.e., colors.

Using a hash function H with a sufficiently large range, we assign a hash value to each singleton color $H(\{r\})$, $r \in \mathcal{R}$. Then, it is possible to form an Abelian group by setting non-singleton colors' hash values to $H(C) = \sum_{r \in C} H(\{r\})$. Since we only compute Parent of disjoint colors, we could obtain $H(C)$ by summing $H(C_1) + H(C_1)$. Thus, $\mathbf{G}$ could be stored in an associative array where we keep $H(C)$ as the key and either $H(C_1)$ or $H(C_2)$ as the value, since one can always compute the other by subtraction. When $C \notin \mathbf{G}$, we simply add it, and return $H(C_1) + H(C_2)$. When $C \in \mathbf{G}$, it could be that it is either already seen due to another k-mer or it is a collision. We check collisions by comparing the value associated with $H(C)$ and $H(C_1)$, $H(C_2)$. Despite being extremely rare, collisions are possible. We handle collisions by adding a dummy reference, i.e., a nonce, to the color C. At the beginning, we ensure that there is no collision among colors corresponding to internal nodes (and singletons) by rehashing.

At the end, in order to avoid storing redundantly large hash values, we simply convert colors to integers by enumerating them (using $\log(|\mathcal{C}|)$ bits) and store them in an array where indexes correspond to color encodings, instead of an associative array.

Derivative of the likelihood function

Given, $u_r, \mathbf{v}_r, \delta, k, h$ and ρ_r, we can compute the derivate of the log-likelihood, Eq. (2), with respect to D as follows:

$$\frac{\mathrm{d}}{\mathrm{d}D}\left(u_r \log\left(P_{\text{miss}}(D;k,h,\delta,\rho_r)\right) + \sum_{d=0}^{\delta} v_{r,d}\left(d\log(D) + (k-d)\log(1-D)\right)\right)$$
$$= \frac{\mathrm{d}}{\mathrm{d}D} u_r \log\left((1-\rho_r) + \rho_r \sum_{d=0}^{k} P_{\text{mutate}}(D;d,k)\left(\mathbb{1}\{d>\delta\} + \mathbb{1}\{d\le\delta\}\left(1 - P_{\text{collide}}(d,k,h)\right)\right)\right)$$
$$+ \sum_{d=0}^{\delta} v_{r,d}\left(d\frac{1}{D} + (d-k)\frac{1}{1-D}\right).$$

For convenience, we define the following function $P_\delta(d)\colon [0,k] \to [0,1]$,

$$P_\delta(d) = \mathbb{1}\{d>\delta\} + \mathbb{1}\{d\le\delta\}\left(1 - \binom{k-h}{d}\Big/\binom{k}{d}\right). \tag{3}$$

where $1 - \binom{k-h}{d}/\binom{k}{d}$ is simply $1 - P_{\text{collide}}(d,k,h)$.

Then, we can substitute $P_{\text{mutate}}(D;d,k)$ with $D^d(1-D)^{(k-d)}\binom{k}{d}$ and compute the derivative of the logarithm,

$$= \frac{\rho_r u_r \left(\sum_{d=0}^{k} D^d(1-D)^{k-d}\binom{k}{d}\left(\frac{d-k}{1-D} + \frac{d}{D}\right)P_\delta(d)\right)}{1-\rho_r+\rho_r\left(\sum_{d=0}^{k} D^d(1-D)^{k-d}\binom{k}{d}P_\delta(d)\right)} + \sum_{d=0}^{\delta} v_{r,d}\left(d\frac{1}{D} + (d-k)\frac{1}{1-D}\right)$$
$$= \frac{\rho_r u_r \left(\sum_{d=0}^{k} D^d(1-D)^{k-d}\binom{k}{d}\left(\frac{d-kD}{D(1-D)}\right)P_\delta(d)\right)}{1-\rho_r+\rho_r\left(\sum_{d=0}^{k} D^d(1-D)^{k-d}\binom{k}{d}P_\delta(d)\right)} + \sum_{d=0}^{\delta} v_{r,d}\left(\frac{d-kD}{D(1-D)}\right).$$

The sign of this derivative is positive at $0+\epsilon$, and negative at $1-\epsilon$, which is required for Brent's method to work and find local optima.

The second derivative is given by

$$
\begin{aligned}
&\frac{\mathrm{d}^2}{\mathrm{d}D^2}\left(u_r \log\left(P_{\text{miss}}(D;k,h,\delta,\rho_r)\right) + \sum_{d=0}^{\delta} v_{r,d}\left(d\log(D) + (k-d)\log(1-D)\right)\right) \\
&= \frac{\mathrm{d}}{\mathrm{d}D}\left(\frac{\rho_r u_r \left(\sum_{d=0}^{k} D^d(1-D)^{k-d}\binom{k}{d}\left(\frac{d-kD}{D(1-D)}\right)P_\delta(d)\right)}{1-\rho_r+\rho_r\left(\sum_{d=0}^{k} D^d(1-D)^{k-d}\binom{k}{d}P_\delta(d)\right)}\right) + \sum_{d=0}^{\delta}\frac{\mathrm{d}}{\mathrm{d}D}\left(v_{r,d}\left(\frac{d-kD}{D(1-D)}\right)\right) \\
&= \frac{\mathrm{d}}{\mathrm{d}D}\left(\frac{\rho_r u_r \left(\sum_{d=0}^{k} D^d(1-D)^{k-d}\binom{k}{d}\left(\frac{d-kD}{D(1-D)}\right)P_\delta(d)\right)}{1-\rho_r+\rho_r\left(\sum_{d=0}^{k} D^d(1-D)^{k-d}\binom{k}{d}P_\delta(d)\right)}\right) + \sum_{d=0}^{\delta} v_{r,d}\left(\frac{d(2D-1)-kD^2}{D^2(1-D)^2}\right) \\
&= \frac{g'(D)f(D)-f'(D)g(D)}{\left(1-\rho_r+\rho_r\left(\sum_{d=0}^{k} D^d(1-D)^{k-d}\binom{k}{d}P_\delta(d)\right)\right)^2} + \sum_{d=0}^{\delta} v_{r,d}\left(\frac{d(2D-1)-kD^2}{D^2(1-D)^2}\right),
\end{aligned}
$$

where

$$
g(D) = \rho_r u_r \left(\sum_{d=0}^{k} D^d(1-D)^{k-d}\binom{k}{d}\left(\frac{d-kD}{D(1-D)}\right)P_\delta(d)\right),
$$

and

$$
f(D) = 1-\rho_r+\rho_r\left(\sum_{d=0}^{k} D^d(1-D)^{k-d}\binom{k}{d}P_\delta(d)\right).
$$

Here, the denominator of the first term is always positive, and the second term, due to P_{match} is non-positive for $D \in (0, 0.5)$. Derivatives of $f(D)$ and $g(D)$ are given by

$$
g'(D) = \rho_r u_r \left(\sum_{d=0}^{k}(1-D)^{k-d}D^d\left(\frac{(d^2+(k-1)kD^2-d\,(1+(k-1)2D))}{D^2(1-D)^2}\right)\binom{k}{d}P_\delta(d)\right),
$$

and

$$
f'(D) = \rho_r\left(\sum_{d=0}^{k} D^d(1-D)^{k-d}\left(\frac{d-kD}{D(1-D)}\right)\binom{k}{d}P_\delta(d)\right).
$$

Thus, $f'(D)g(D)$ is always non-negative:

$$
u_r\rho_r^2\left(\sum_{d=0}^{k} D^d(1-D)^{k-d}\left(\frac{d-kD}{D(1-D)}\right)\binom{k}{d}P_\delta(d)\right)^2.
$$

It suffices to show $g'(D)f(D) \leq 0$ to prove the concavity of the log-likelihood. Notice that we can simplify the entire second derivative without changing the sign by multiplying all terms with $D^2(1-D)^2$. Furthermore, since $f(D) > 0$ for $\rho_r \in [0,1]$ and $D \in (0, 0.5)$, we can focus on the

following quantity:

$$\sum_{d=0}^{k}(1-D)^{k-d}D^d\left(d^2+(k-1)kD^2-d\left(1+(k-1)2D\right)\right)\binom{k}{d}P_\delta(d),$$

which is equal to 0 without $P_\delta(d)$:

$$\sum_{d=0}^{k}(1-D)^{k-d}D^d\left(d^2+(k-1)kD^2-d\left(1+(k-1)2D\right)\right)\binom{k}{d}=0.$$

This can be shown by

$$\begin{aligned}
&\sum_{d=0}^{k}(1-D)^{k-d}D^d\left(d^2+(k-1)kD^2-d\left(1+(k-1)2D\right)\right)\binom{k}{d}\\
&=\sum_{d=0}^{k}\left(d^2(1-D)^{k-d}D^d\binom{k}{d}\right)-((2k-2)D+1)\sum_{d=0}^{k}\left(d(1-D)^{k-d}D^d\binom{k}{d}\right)\\
&+k(k-1)D^2\sum_{d=0}^{k}\left((1-D)^{k-d}D^d\binom{k}{d}\right)\\
&=\mathbb{E}(\mathrm{X}^2)+(D(2k-2)+1)\,\mathbb{E}(\mathrm{X})+k(k-1)D^2(1)\\
&=\left(k^2D^2+(1-D)kD\right)+(D(2k-2)+1)\,(kD)+k(k-1)D^2=0,
\end{aligned}$$

where $X\sim\text{Binomial}(k,D)$.

Finally, to conclude that $g'(D)\leq 0$, we need to show the following inequality holds after including $P_\delta(d)$ back:

$$\sum_{d=0}^{\delta}(1-D)^{k-d}D^d\left(d^2+(k-1)kD^2-d\left(1+(k-1)2D\right)\right)\binom{k-h}{d}\geq 0. \tag{4}$$

This inequality is not true for all choices of δ, h and k, but the default parameter values k=29, h=14, δ=4 satisfy it for $D\in(0,0.5)$

Limitations of APPLES

Reads come from various parts of the genome, and the rates of evolution and, thus, branch lengths change across the genome. For the least square method APPLES [63] to work well, distances from query to references should be in the same scale as distances among references. This observation was one of the main insights from the original papers. Having consistent estimates of branch length necessitates recomputing distances among references for each read, which is possible with our data structure. However, it will make placement too slow as it would need $O(kn^2)$ computations for k queries and n references, instead of the desired $O(kn)$, provided by krepp. Ignoring changes in rates reduces accuracy and is not a viable option [63].

Software versions and commands used

Here, we provide the exact commands that we used to run external tools and krepp throughout our experiments, together with their version information.

Genomic distance estimation using Mash

We used Mash (version 2.3) to estimate genomic distances. To create a Mash sketch from a genome and then use it to estimate genomic distance, we used the below commands.

```
mash sketch -k 29 -s 100000 -p $NUM_THREADS -o $SKETCH_FILE $INPUT_FASTA
mash dist $SKETCH_FILE1 $SKETCH_FILE2
```

Short read simulation using ART

We simulated short reads with length L and coverage c, with the default error and quality profiles of Illumina HiSeq 2500 using ART [76] (version 2.5.8 – single read mode) with the command below.

```
art_illumina -ss HS25 -l L -f c -na -s 10 -i $INPUT_FASTA -o $OUTPUT_FASTQ
```

Downsampling reads using `seqtk`

To subsample read collection down to a specified number of reads, denoted by n here, we used `seqtk` [77] (version 1.3r106) with the command below.

```
seqtk sample -s 150 $INPUT_FASTQ n > $OUTPUT_FASTQ
```

Read alignment using bowtie2

We used bowtie2 [36] (v2.4.1) for short read alignment. To construct an index from all genomes combined in a single FASTA file (`$INPUT_FASTA`), we used the command below.

```
bowtie2-build --large-index --threads 32 $INPUT_FASTA $OUTPUT_DATABASE
```

Given an index (`$INPUT_DATABASE`), we used the following alignment configuration to generate SAM files for all alignments.

```
bowtie2 -p $NUM_THREADS -x $INPUT_DATABASE -t -q -U $QUERY_FASTQ \
   --xeq --very-sensitive --all -S $OUTPUT_SAMFILE
```

We postprocessed `$OUTPUT_SAMFILE` to compute Hamming distance for each alignment using a custom script provided at `https://github.com/bo1929/shared.krepp/blob/main/scripts/postprocess_sam.py`.

Distance estimation and phylogenetic placement using krepp

The version of krepp we presented in this work is v0.4.5. Given a mapping between reference IDs (leaves of the tree) and paths, together with an optional guide tree, krepp builds an index from k-mers of the reference genomes in batches, and the total number of batches is specified by the option `-m`. Each batch is built with a separate command using the option `-r` as shown in the command below.

```
krepp index -o $OUTPUT_INDEX -i $INPUT_FILE -t $BACKBONE_NEWICK --no-frac \
    --num-threads $NUM_THREADS -k k -w w -h h-m $NUM_BATCHES -r $BATCH_INDEX
```

For WoL indexes, we set $k = 29$, $w = 35$, and $h = 14$. For larger RefSeq snapshots, we opt for $k = 30$, $w = 37$, and $h = 14$.

Once the index is built, one can query reads against it for either distance estimation or phylogenetic placement using the following commands, setting $\delta = 4$:

```
krepp dist -i $INPUT_INDEX -q $QUERY_FASTQ -hdist-th δ \
--num-threads $NUM_THREADS -o $OUTPUT_DISTANCES
krepp place -i $INPUT_INDEX -q $QUERY_FASTQ -hdist-th δ \
--num-threads $NUM_THREADS -o $OUTPUT_JPLACE
```

BIOM table construction using Woltka

We converted mappings of short reads to BIOM tables, which are simply matrices of counts of observations on a per-sample basis, using Woltka (version 0.1.7). In particular, we ran `woltka classify --no-demux --digits 5 -i $INPUT_MAPPINGS -o $OUTPUT_BIOM`, where `$INPUT_MAPPINGS` is a directory containing tab-separated files for read ID to subject (e.g., reference genome, internal node, taxon) mappings for each sample. To filter low-abundance subjects, we used `woltka filter -i $INPUT_BIOM -o $OUTPUT_BIOM --min-percent 0.01`.

Genome-wide phylogenetic placement using App-SpaM

We used App-SpaM [19] (v1.03) to place short reads with respect to reference sequences, combined in a single FASTA file (`$INPUT_FASTA`), on a given backbone tree (`$BACKBONE_NEWICK`) by running the command below.

```
appspam --threads $NUM_THREADS -s $INPUT_FASTA -t $BACKBONE_NEWICK -q $QUERY_FASTA
```

Marker-based phylogenetic placement using EPA-ng

For maximum likelihood placement, the branch lengths of the backbone tree are re-estimated using RAxML [94] (v1.2.2) using the command below.

```
raxml-ng -evaluate --threads $NUM_THREADS --msa $REFERENCE_MSA \
   --tree $BACKBONE_NEWICK --prefix $OUTPUT_PATH/mltree \
   --model GTR+G+F --force --blopt nr_safe
```

Using the output tree from the above command, we used EPA-ng (version 0.3.8) as shown below to place queries.

```
epa-ng --ref-msa $REFERENCE_MSA --tree $BACKBONE_NEWICK --query $QUERY_FASTA \
   --model $OUTPUT_PATH/mltree.raxml.bestModel -w $OUTPUT_DIR \
   --verbose --redo -T $NUM_THREADS
```

Rarefication and psedo-F calculation using QIIME2

We computed separation statistics and distances between sample pairs using QIIME2 [80] (version 2024.5.0), and specifically its diversity plugin (version 2024.5.1). After performing rarefication at sampling depth S (set to $100,000$ and $6,550$ for human microbiome and Earth's microbiome analyses, respectively), to compute weighted UniFrac distance and Bray-Curtis dissimilarity, we used used `core-metrics-phylogenetic` command.

```
qiime diversity core-metrics-phylogenetic --i-table $FEATURE_TABLE_QZA \
   --i-phylogeny $BACKBONE_QZA --m-metadata-file $METADATA_FILE \
   --p-sampling-depth S --p-ignore-missing-samples --output-dir $OUTPUT_DIR
```

Next, once the distance matrices are obtained, we used `beta-group-significance` command to compute pseudo-F statistics for all groupings as shown below.

```
qiime diversity beta-group-significance --i-distance-matrix $DISTANCE_MATRIX_QZA \
   --m-metadata-file $METADATA_FILE --m-metadata-column $METADATA_COLUMN \
   --o-visualization $OUTPUT_QZV --p-permutations 1000
```
